# Endothelial cells require functional FLVCR1a during developmental and adult angiogenesis

**DOI:** 10.1007/s10456-023-09865-w

**Published:** 2023-01-11

**Authors:** Sara Petrillo, F. De Giorgio, F. Bertino, F. Garello, V. Bitonto, D. L. Longo, S. Mercurio, G. Ammirata, A. L. Allocco, V. Fiorito, D. Chiabrando, F. Altruda, E. Terreno, P. Provero, L. Munaron, T. Genova, A. Nóvoa, A. R. Carlos, S. Cardoso, M. Mallo, M. P. Soares, E. Tolosano

**Affiliations:** 1 Department of Molecular Biotechnology and Health Sciences, Molecular Biotechnology Center (MBC) “Guido Tarone”, University of Torino, Via Nizza, 52, 10126 Turin, Italy; 2 Institute of Biostructures and Bioimaging (IBB), Italian National Research Council (CNR), Via Nizza, 52, 10126 Turin, Italy; 3 Department of Molecular Biotechnology and Health Sciences, and GenoBiToUS, Genomics and Bioinformatics Service, University of Torino, Turin, Italy; 4 Center for Translational Genomics and Bioinformatics, San Raffaele Scientific Institute IRCCS, Milan, Italy; 5 Department of Life Sciences and Systems Biology, University of Torino, Via Accademia Albertina 13, 10123 Turin, Italy; 6 Instituto Gulbenkian de Ciência, Oeiras, Portugal

**Keywords:** Angiogenesis, FLVCR1, Endothelial cell, Tumor endothelial cell

## Abstract

The Feline Leukemia Virus Subgroup C Receptor 1a (FLVCR1a) is a transmembrane heme exporter essential for embryonic vascular development. However, the exact role of FLVCR1a during blood vessel development remains largely undefined. Here, we show that FLVCR1a is highly expressed in angiogenic endothelial cells (ECs) compared to quiescent ECs. Consistently, ECs lacking FLVCR1a give rise to structurally and functionally abnormal vascular networks in multiple models of developmental and pathologic angiogenesis. Firstly, zebrafish embryos without FLVCR1a displayed defective intersegmental vessels formation. Furthermore, endothelial-specific *Flvcr1a* targeting in mice led to a reduced radial expansion of the retinal vasculature associated to decreased EC proliferation. Moreover, *Flvcr1a* null retinas showed defective vascular organization and loose attachment of pericytes. Finally, adult neo-angiogenesis is severely affected in murine models of tumor angiogenesis. Tumor blood vessels lacking *Flvcr1a* were disorganized and dysfunctional. Collectively, our results demonstrate the critical role of FLVCR1a as a regulator of developmental and pathological angiogenesis identifying FLVCR1a as a potential therapeutic target in human diseases characterized by aberrant neovascularization.

**Supplementary Information:**

The online version contains supplementary material available at 10.1007/s10456-023-09865-w.

## Introduction

An extensive and finely organized three-dimensional micro vascular tree ensures nutrients and oxygen delivery to peripheral tissues. To this end, blood vessels develop during early embryonic stages through a complex coordination of vasculogenesis and angiogenesis to supply the increasing demand of the developing embryo [1]. In the mouse embryo, developmental angiogenesis occurs mostly between embryonic days (E) 8.0 and 10.0, during which primitive endothelial tubes undergo extensive remodeling giving rise to a hierarchically branched, highly organized vascular network [2–5]. In adults, endothelial cells (ECs) are mostly quiescent [6]. However, they still retain the ability to reactivate and initiate angiogenesis, such as observed at steady state during menstrual cycle and pregnancy as well as under pathological conditions, in response to tissue injury [1, 7]. Moreover, neo-angiogenesis also plays a critical role in major pathological conditions such as ocular neovascularization diseases and cancer [8, 9]. In such conditions, aberrant pro-angiogenic signaling dysregulates angiogenesis leading to irregular, tortuous, and low perfused blood vessels [10]. Importantly, these vascular alterations have a prominent role in the tumor context, favoring tumor growth and cancer cells dissemination [11].

The Feline Leukemia Virus Subgroup C Receptor 1a (FLVCR1a) is an evolutionary conserved cell membrane heme exporter critically involved in vascular development [12, 13]. FLVCR1a exports de novo synthetized heme and sustains heme synthesis by preventing heme-mediated inhibition of ALAS1, the rate limiting enzyme of heme biosynthesis [14]. Endothelial-specific *Flvcr1a* null mice display proper vasculogenesis at early stage of development but develop severe vessel defects starting from E11.5 and resulting in widespread hemorrhages and intrauterine death. Flvcr1a-deficient ECs show several structural abnormalities including cytosolic vacuoles, enlarged endoplasmic reticulum (ER), and damaged mitochondria [12]. While highlighting a pivotal role of FLVCR1a in the vascular endothelium, these studies did not clarify whether FLVCR1a is required for angiogenic endothelial cells (aECs) during blood vessels formation or maintenance of quiescent endothelial cells (qECs) in adult vascular beds. Here, we used several models to investigate the role of FLVCR1a during both physiological and pathological angiogenesis as well as in qECs.

We found that FLVCR1a is preferentially expressed in angiogenic endothelium, where it is required for proper developmental angiogenesis and adult neo-angiogenesis whereas it is dispensable in qECs.

## Results

### FLVCR1a is highly expressed in angiogenic endothelial cells

**Fig. 1:**
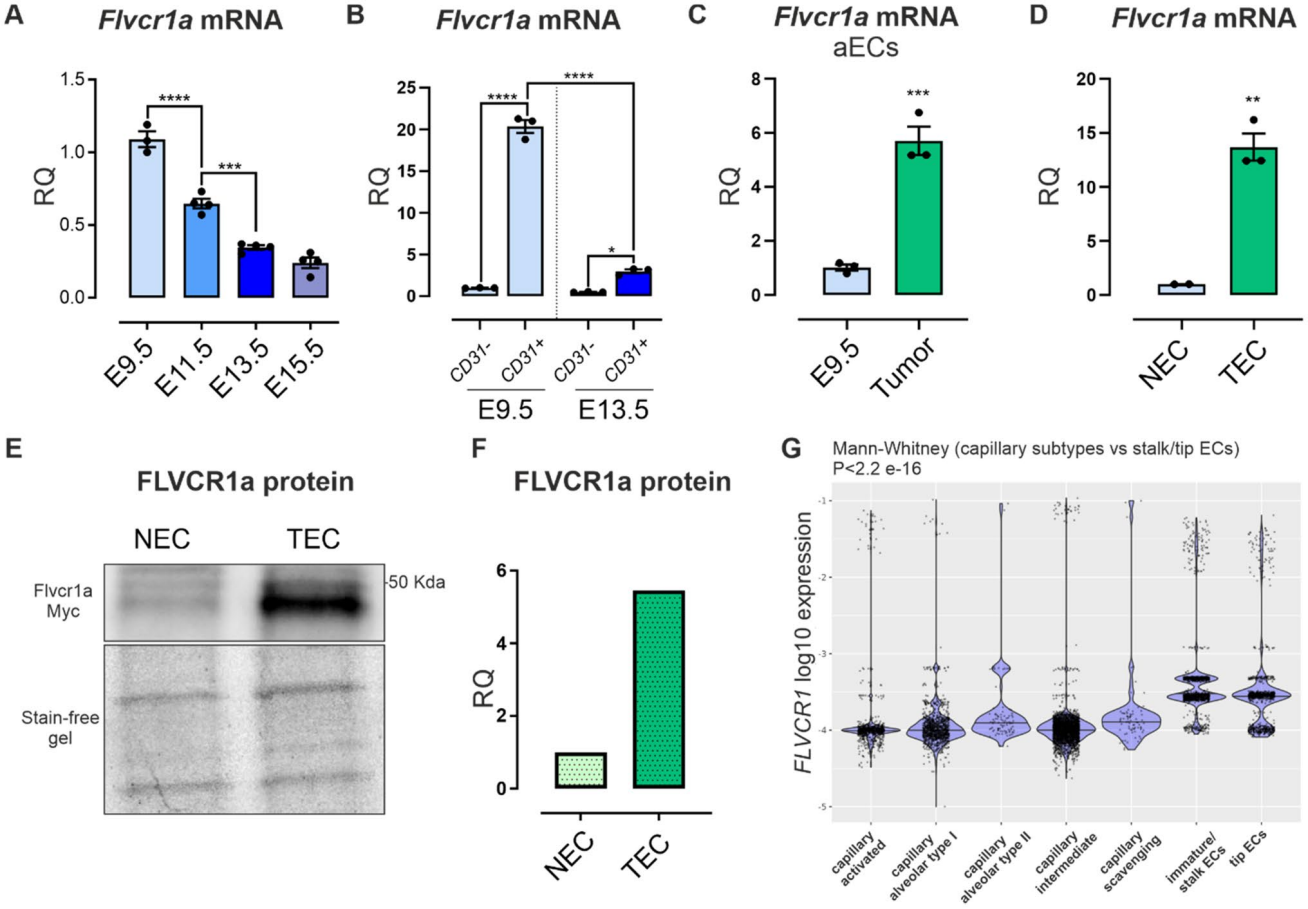
FLVCR1a expression is enhanced in angiogenic endothelial cells (aECs). **A** qRT-PCR analysis showing *Flvcr1a* mRNA levels in wild-type mouse embryos at different developmental stages (i.e., embryonic day (E) 9.5, 11.5, E13.5, E15.5). *n* = 4. **B** qRT-PCR analysis showing *Flvcr1a* transcript levels in embryonic ECs (CD31+) and non-endothelial fractions (CD31−) collected from mouse embryos at E9.5 and E13.5. *n* = 3. **C** qRT-PCR analysis showing *Flvcr1a* mRNA levels in embryonic angiogenic ECs (E9.5) and tumor-associated ECs (TECs) from Lewis Lung Carcinoma Cells (LLC)-xenografts. **D** qRT-PCR analysis showing *Flvcr1a* transcript levels in mouse ECs isolated from adult lung and LLC tumors. **E**, **F** Representative Western Blot analysis (**E**) and quantification (**F**) of FLVCR1a-myc-tagged protein expression in murine ECs from adult lung and LLC tumor. **G** *FLVCR1a* expression levels in human qECs (capillary ECs) and aECs (immature/stalk and tip cells) isolated from lung adenocarcinoma. Data were taken from public available database. *aECs* angiogenic ECs, *NEC/TEC* Normal/Tumor ECs, *RQ* Relative Quantification. Data are representative of at least 3 independent experiments and are expressed as mean ± SEM. *p < 0.05; **p < 0.01; ***p < 0.001; ****p < 0.0001. For statistical analyses, ordinary one-way ANOVA test with Tukey’s multiple comparisons (**A**, **B**), parametric unpaired t test (**C**, **D**, **F**), and Mann–Whitney test (**G**) were used

To elucidate the relevance of FLVCR1a during embryonic vascular development, *Flvcr1a* mRNA expression was analyzed at different murine embryonic stages (E) by reverse transcriptase-quantitative polymerase chain reaction (RT-qPCR). As illustrated in Fig. 1A, *Flvcr1a* mRNA was highly expressed at E9.5 and underwent a progressive decline during later developmental stages (i.e., E11.5, E13.5, E15.5). To understand the contribution of the endothelial lineage to *Flvcr1a* expression fluctuations during embryonic development, ECs were isolated from wild-type embryos through immunomagnetic separation. *Flvcr1a* expression was enriched in ECs (CD31+), compared to non-ECs (CD31-) at both E9.5 and E13.5 (Fig. 1B), supporting a prominent role of FLVCR1a in ECs during embryonic development. Moreover, endothelial *Flvcr1a* expression displayed a drastic drop off at E13.5 (Fig. 1B). Given that major angiogenic events in mouse embryos occur between E8.0 and E10.0, these results highlight the relevance of endothelial FLVCR1a in the temporal frame mostly associated with developmental angiogenesis, suggesting that this heme exporter becomes dispensable once the vascular network has reached a remodeled structure.

To address the additional requirement of FLVCR1a in pathologic angiogenesis, tumor-associated ECs (TECs) were isolated from Lewis Lung Carcinoma (LLC) subcutaneous xenografts, and *Flvcr1a* expression was analyzed in TECs compared to embryonic aECs from E9.5 embryos. *Flvcr1a* expression was remarkably elevated in TECs, when compared to aECs (Fig. 1C). This indicates that FLVCR1a is required not only during physiologic angiogenesis but also during aberrant angiogenesis associated to tumor development. Furthermore, *Flvcr1a* transcript levels were much higher in angiogenic TECs than in qECs from the adult lung, supporting a fundamental role of FLVCR1a in aECs (Fig. 1D). Analyses of FLVCR1a protein expression in a knock-in mouse strain expressing a myc-tagged FLVCR1 (Fig. S1) showed an increased FLVCR1a protein expression in TECs compared to NECs isolated from LLC tumors and lungs, respectively (Fig. 1E, F). Finally, *FLVCR1a* expression was analyzed in human samples by mining publicly available data obtained from single-cell RNA sequencing (scRNAseq) analyses performed on ECs isolated from human lung cancer. *FLVCR1a* expression in aECs, i.e., tip and stalk/immature cells of the tumor, was compared to that in qECs isolated from the peri-tumoral tissue (i.e., capillary alveolar ECs). Consistent with the analysis on mouse ECs (Fig. 1D), *FLVCR1a* expression was higher in human tumor-associated aECs as compared to matched qECs (Fig. 1G).

Taken together, these data demonstrate that FLVCR1a is highly expressed in ECs undergoing angiogenesis, whereas its expression switches off when the vascular network is established.

### FLVCR1a is required for developmental angiogenesis

**Fig. 2:**
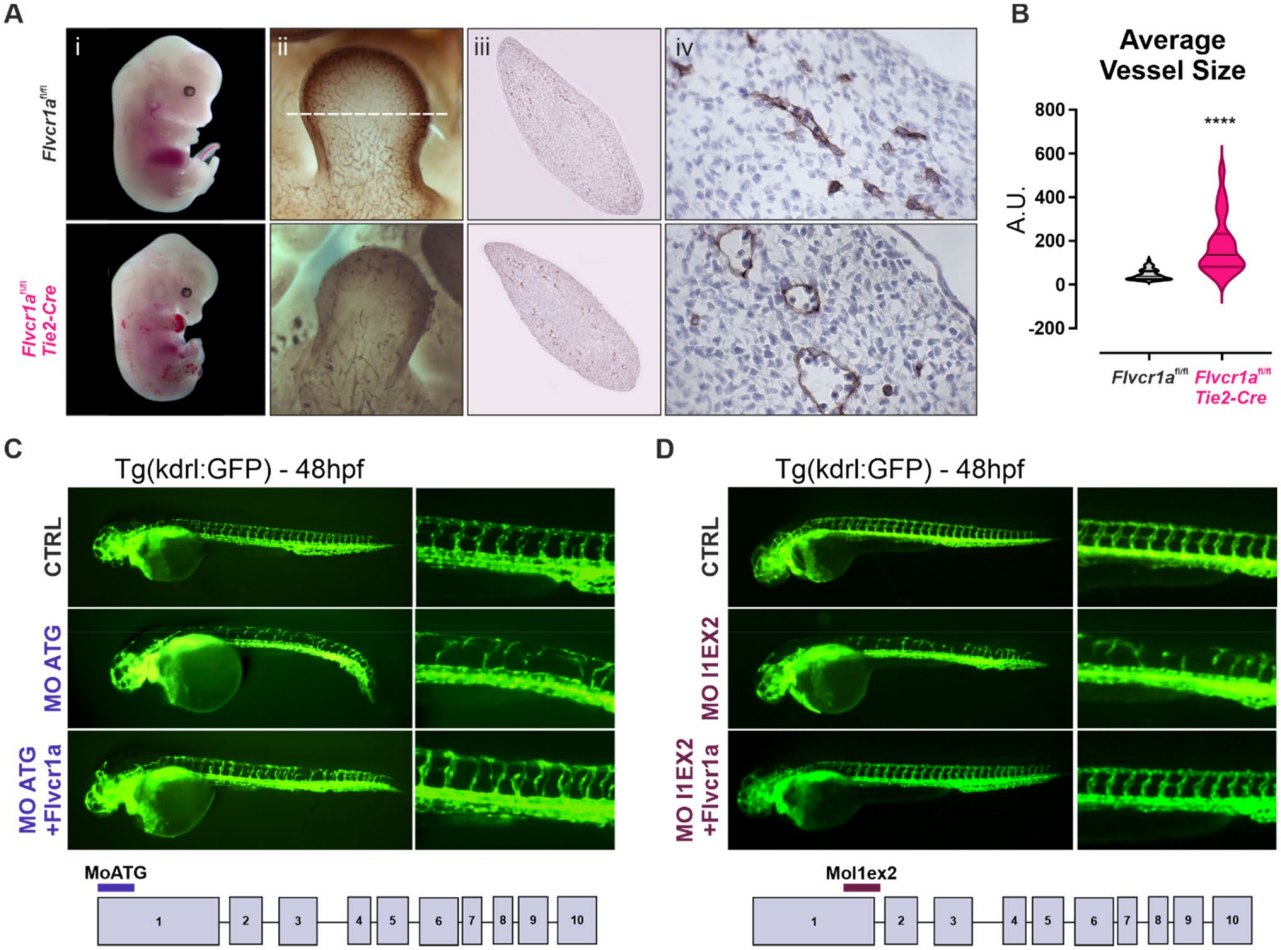
*Flvcr1a* targeting impairs developmental angiogenesis in mouse and zebrafish embryos. **A**(i) Phenotype of E13.5 control (*Flvcr1a*^fl/fl^) and *Flvcr1a* endothelial-specific constitutive knockout (*Flvcr1a*^fl/fl^;*Tie2*-Cre) mouse embryos. (ii) Whole-mount staining with CD31/Pecam-1 antibody showing the embryonic vasculature in E11.5 embryos. Enlarged pictures of forelimbs are shown. White dashed line refers to the transverse section displayed in panels iii-iv. (iii-iv) Transverse sections of E11.5 forelimb from *Flvcr1a*^fl/fl^ and *Flvcr1a*^fl/fl^;*Tie2*-Cre embryos stained with CD31/Pecam-1 antibody. **B** Quantification of the average vessel size in *Flvcr1a*^fl/fl^ and *Flvcr1a*^fl/fl^;*Tie2*-Cre limbs transverse sections shown in (iii). **C**, **D** Photographs of transgenic (Tg) kdrl:GFP zebrafish embryos injected with CTRL, MO-ATG, or MOl1EX2 morpholinos alone or with Flvcr1a cRNA. Images were collected at 48 hpf. Magnification of intersegmental vessels in controls, morphants and rescued embryos, are shown. ****p < 0.0001. For statistical analyses, parametric unpaired *t* test was used. *A.U.* arbitrary units, *MO* morpholino, *hpf* hours post fertilization

Consistent with previously reported, endothelial-specific deletion of *Flvcr1a* in mice caused embryonic lethality associated to a severe hemorrhagic phenotype [12] (Fig. 2A(i)). Mutant embryos displayed defective embryonic angiogenesis characterized by an altered and enlarged microvessels morphology starting from E11.5 (Fig. 2A(ii–iv), B). To investigate whether this FLVCR1a function in ECs was conserved among vertebrates, two different morpholinos were used to specifically target *flvcr1a* mRNA in zebrafish embryos. As observed in mice, angiogenesis was severely compromised in zebrafish morphants. In particular, the organization of intersegmental vessels was completely lost in Flvcr1a morphants at 48 h (h) post fertilization (Fig. 2C, D). Moreover, *flvcr1a* cRNA injection in zebrafish morphants fully rescued the angiogenic defects (Fig. 2C, D). Notably, these findings agree with previous data showing that zebrafish Flvcr1a morphants display hemorrhages [15]. These models of FLVCR1a deficiency confirm the conserved role of the transporter in the formation of a functional vascular network during embryogenesis.

**Fig. 3:**
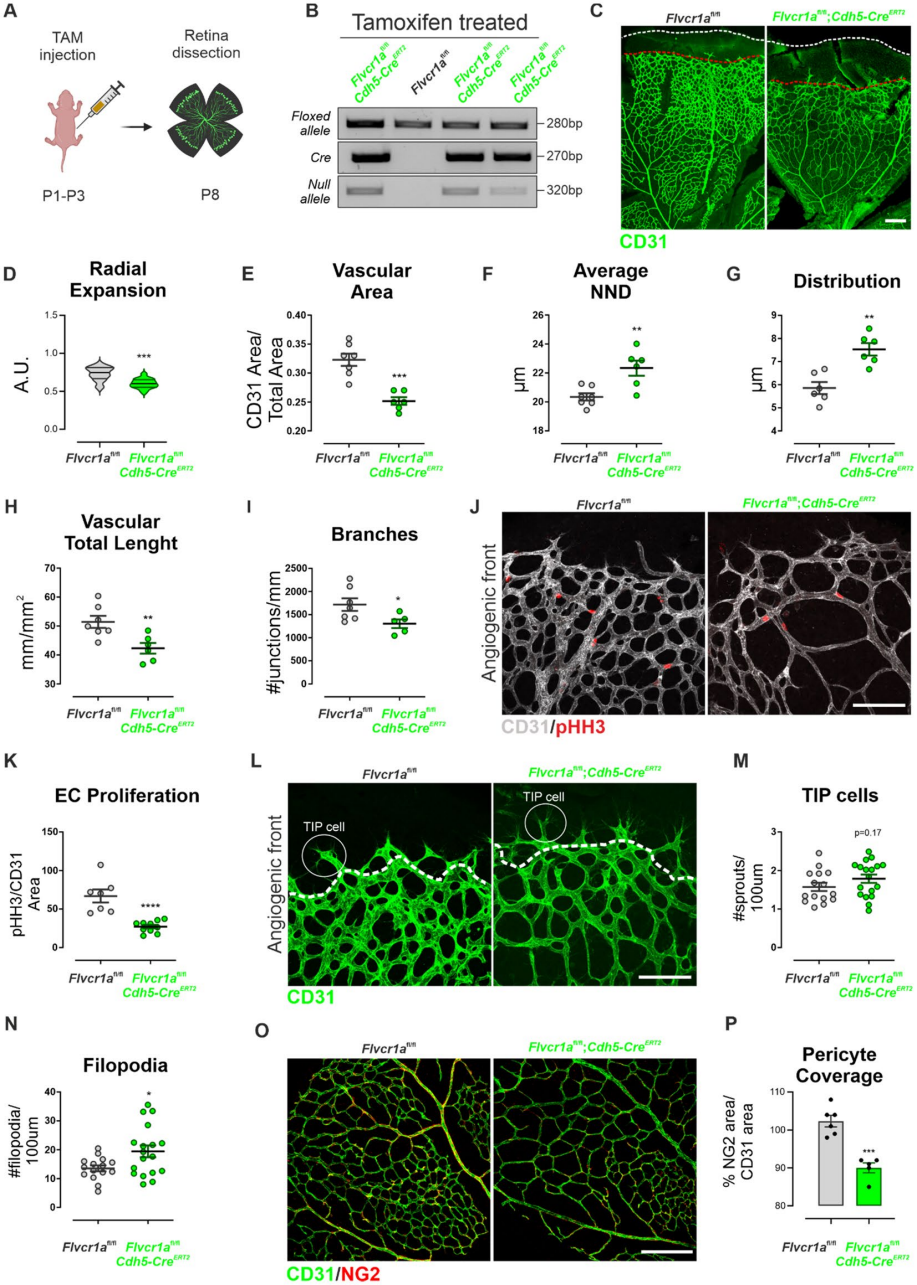
Retinal angiogenesis is compromised in endothelial *Flvcr1a*-null mice. **A** Schematic representation of tamoxifen (TAM) treatment and retina dissection in *Flvcr1a*;*Cdh5-Cre*^ERT2^ and *Flvcr1a* control mice. Briefly, pups were injected with TAM into the stomach for 3 consecutive days (P1–P3) to induce *Flvcr1a* gene deletion in the developing retinal vasculature. Retinas were isolated at P8 to analyze angiogenesis. **B** Representative PCR products from DNA analysis on *Flvcr1a*^fl/fl^ and *Flvcr1a*^fl/fl^;*Cdh5-Cre*^ERT2^ pups after tamoxifen treatment. Deletion of the first exon of *Flvcr1a* gene mediated by Cre recombinase gave rise to a band referred to as ‘‘null allele.’’ Specific primers were used to amplify *Cre* recombinase (270 bp), *Flvcr1a* floxed (280 bp), and *Flvcr1a* null (320 bp) alleles. **C** Whole-mount CD31 staining (green) of P8 retinal primary vascular plexus from *Flvcr1a*^fl/fl^ and *Flvcr1a*^fl/fl^;*Cdh5-Cre*^ERT2^ mice. Scale bar: 200 µm. **D**–**I** Quantification of vascular parameters of *Flvcr1a*^fl/fl^ and *Flvcr1a*^fl/fl^;*Cdh5-Cre*^ERT2^ P8 retinas, i.e., **D** radial expansion, **E** vascular area, **F** average nearest neighbor distance (NND), **G** vessel distribution variability, **H** vascular total length, **I** number of branches. **J** CD31 (gray)/pHH3 (red) double staining of *Flvcr1a*^fl/fl^ and *Flvcr1a*^fl/fl^;*Cdh5-Cre*^ERT2^ P8 retinas, and **K** quantification of pHH3^+^ ECs. Scale bar: 100 µm. **L** Higher magnification of the angiogenic front in *Flvcr1a*^fl/fl^ and *Flvcr1a*^fl/fl^;*Cdh5-Cre*^ERT2^ P8 retinas stained with CD31 antibody to highlight the vasculature. Tip cells are white circled. Scale bar: 100 µm. **M** Tip cells and **N** filopodia quantification is shown. **O** Whole-mount staining of *Flvcr1a*^fl/fl^ and *Flvcr1a*^fl/fl^;*Cdh5-Cre*^ERT2^ P8 retinas with CD31 (green) and NG2 (red) antibodies to highlight ECs and pericyte, respectively. Scale bar: 200 µm. **P** Quantification of pericyte coverage as % ratio of NG2 area and CD31 area. Data are representative of at least 3 independent experiments and are expressed as mean ± SEM. *p < 0.05; **p < 0.01; ***p < 0.001; ****p < 0.0001; For statistical analyses ,an unpaired Student’s t test was used. *TAM* tamoxifen, *P* post-natal day, *bp* base pairs, *A.U.* arbitrary unit

Another well-established model of developmental angiogenesis is the mouse retina. Unlike humans, the initial outgrowth of the retinal vasculature from the optic nerve toward the periphery starts around post-natal day (P) 3. This is followed by a rapid and continuous radial expansion of the vasculature until it covers the whole retinal area by the age of P10 [16]. To test the relevance of FLVCR1a in sprouting angiogenesis, *Flvcr1a* expression was specifically abolished in post-natal ECs. To this purpose, vascular endothelial (VE)-cadherin (PAC)-Cre^ERT2^ mice [17] were crossed with *Flvcr1a*^lox/lox^ mice and the progenies were treated with tamoxifen at P1 to abolish *Flvcr1a* expression specifically in ECs (Fig. 3A, B). Then, neonatal retinas were dissected at P8 and immunofluorescence (IF) analysis was performed. In agreement with the other models of developmental angiogenesis, an irregular and underdeveloped primary vascular plexus was found in *Flvcr1a*;*Cdh5-Cre*^ERT2^ mice at P8 (Fig. 3C). The radial expansion of the primary plexus vascular network was delayed in *Flvcr1a*;*Cdh5-Cre*^ERT2^ retinas (Fig. 3D). Also, a series of morphological parameters describing how vessels organize and arrange in the space were considered. This analysis showed that *Flvcr1a*;*Cdh5-Cre*^ERT2^ retinas displayed defective vascular organization, as indicated by quantification of the vascular area fraction, average nearest neighbor distance (NND), hierarchical distribution of vessels, vascular total length, and number of branches (Fig. 3E–I). To elucidate if a defect in EC proliferation could account for this phenotype, phosphorylated histone H3 (pHH3) was analyzed in P8 retinas. *Flvcr1a*;*Cdh5-Cre*^ERT2^ retinas showed a reduced number of mitotic cells both at the angiogenic front and close to the central plexus, indicating that defective angiogenesis was partly due to reduced EC proliferation (Fig. 3J–K). This result is consistent with previously published data showing that *FLVCR1a* knockdown decreases human EC proliferation in vitro [12]. Further examination of P8 *Flvcr1a*;*Cdh5-Cre*^ERT2^ retinas revealed a similar number of tip cells at the angiogenic front (Fig. 3L–M) along with a slight increase in the number of elongated filopodia (Fig. 3N). Finally, the pericyte coverage of retinal vessels was measured by IF staining with the pericyte’s marker chondroitin sulfate proteoglycan (NG2). As shown in Fig. 3O–P, the *Flvcr1a*;*Cdh5-Cre*^ERT2^ retinal vasculature displayed a reduced number of pericytes associated to blood vessels, indicating impaired pericyte recruitment.

Taken together these data demonstrate that FLVCR1a is required for post-natal retinal vascularization, further supporting its relevant role in developmental angiogenesis.

### FLVCR1a is required for pathologic neo-angiogenesis in the adulthood

**Fig. 4:**
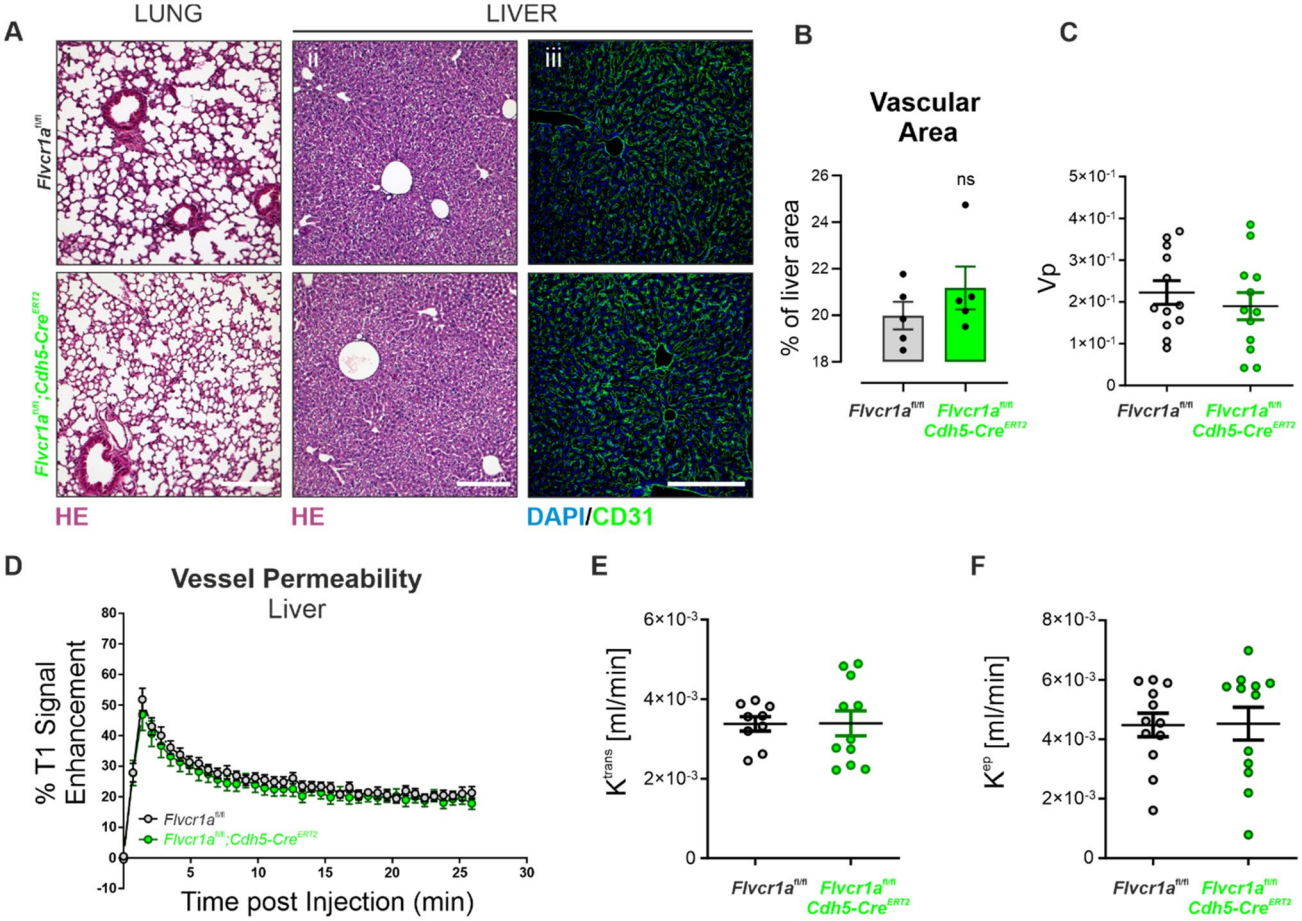
Endothelial *Flvcr1a* is dispensable in adult healthy tissues. **A** Representative histological sections of lung and liver tissue from *Flvcr1a*^fl/fl^ and *Flvcr1a*^fl/fl^;*Cdh5-Cre*^ERT2^ mice. (i, ii) Tissue sections stained with hematoxylin and eosin (HE) to look at the cellular morphology. Scale bar: 200 µm. (iii) Confocal images of liver sections from *Flvcr1a*^fl/fl^ and *Flvcr1a*^fl/fl^;*Cdh5-Cre*^ERT2^ stained with CD31 antibody (green) and DAPI (blu) to look at vascular network organization. Scale bar: 200 µm. **B** Liver vascular area quantification expressed as % of liver area positive to CD31. **C**–**F** Gadolinium-based DCE-MRI analysis to evaluate (**C**) the total vascular volume (Vp) and **D**–**F** vessel permeability in *Flvcr1a*^fl/fl^ and *Flvcr1a*^fl/fl^;*Cdh5-Cre*^ERT2^ livers. HE, hematoxylin/eosin DCE-MRI, Dynamic Contrast-Enhanced Magnetic Resonance Imaging. Data are representative of at least 3 independent experiments and are expressed as mean ± SEM. **p < 0.01; ***p < 0.001; *ns* not significant. For statistical analyses, an unpaired Student’s t test was used

To elucidate the role of FLVCR1a in adulthood, 6-week-old *Flvcr1a*;*Cdh5-Cre*^ERT2^ mice were treated with tamoxifen to delete FLVCR1a specifically in the adult vasculature. Polymerase chain reaction (PCR) analysis showed that the *Flvcr1a* alleles were correctly recombined following tamoxifen treatment (Fig. S1A), while RT-qPCR confirmed that *Flvcr1a* mRNA was reduced in ECs isolated from lung and liver of tamoxifen-treated *Flvcr1a*;*Cdh5-Cre*^ERT2^ mice (Fig. S1B, C). No alterations in lung (Fig. 4A(i)), liver (Fig. 4A(ii)), and kidney (Fig. S1D) parenchyma were found in tamoxifen-treated *Flvcr1a*;*Cdh5-Cre*^ERT2^ mice. Furthermore, the vascular organization within the liver was not affected by *Flvcr1a* deletion in adult mice, as indicated by IF staining for the endothelial marker CD31 (Fig. 4A(iii)). The functionality of healthy organs’ vasculature was assessed through dynamic contrast-enhanced magnetic resonance imaging (DCE-MRI) performed with a gadolinium-based contrast agent injected into the tail vein. The kinetics of the contrast agent were analyzed, while it passed throughout the liver (Fig. 4C–F) and muscle (Fig. S1E–H) vasculature, showing no alteration in the total vascular volume (V_p_) and vessels permeability (K^trans^). These data overall indicate that endothelial FLVCR1a is dispensable in adult healthy tissues characterized by fully formed and mature vascular beds.

**Fig. 5:**
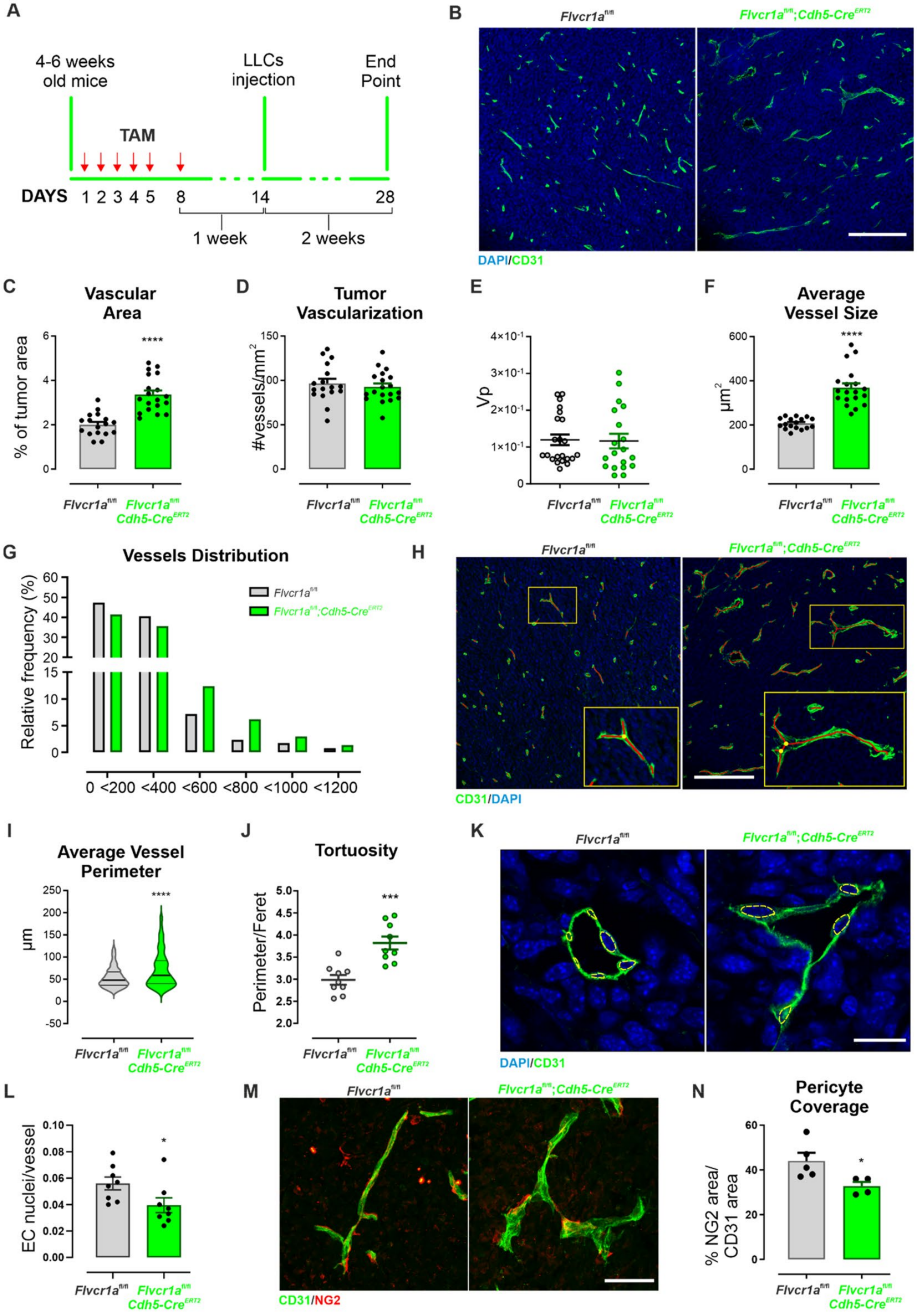
Endothelial *Flvcr1a* deficiency impairs tumor angiogenesis. **A** Schematic representation of tamoxifen (TAM) treatment in *Flvcr1a*^fl/fl^;*Cdh5-Cre*^ERT2^ and *Flvcr1a*^fl/fl^ control mice. **B** Confocal images of CD31 (green) and DAPI (blu)-stained tumor sections from *Flvcr1a*^fl/fl^ and *Flvcr1a*^fl/fl^;*Cdh5-Cre*^ERT2^ to highlight the tumor vasculature. **C** Tumor vascular area quantification as % CD31 of tumor area. Scale bar: 200 µm. **D** Tumor vascularization expressed as number of vessels/mm^2^. **E** Gadolinium-based DCE-MRI analysis to measure tumor vascular volume (Vp) in *Flvcr1a*^fl/fl^ and *Flvcr1a*^fl/fl^;*Cdh5-Cre*^ERT2^ animals. **E**–**G** Quantification of (E) average vessel size, **F** vessel area distribution and **G** average vessel perimeter in *Flvcr1a*^fl/fl^ and *Flvcr1a*^fl/fl^;*Cdh5-Cre*^ERT2^ tumors. **H** Representative images showing the ImageJ “skeletonize tool” used to infer network descriptive parameters (e.g., vessel perimeter, Feret’s diameter) on tumor sections. **I**–**J** Histograms showing quantification of **I** average vessel perimeter and **J** vessel tortuosity (expressed as perimeter/Feret’s diameter) in *Flvcr1a*^fl/fl^ and *Flvcr1a*^fl/fl^;*Cdh5-Cre*^ERT2^ tumor sections. **K** High magnification of CD31 (green) and DAPI (blu)-stained *Flvcr1a*^fl/fl^ and *Flvcr1a*^fl/fl^;*Cdh5-Cre*^ERT2^ tumor vessels. ECs nuclei are comprised in yellow dashed lines. Scale bar: 20 µm. **L** Quantification of average number EC nuclei per vessel perimeter in *Flvcr1a*^fl/fl^ and *Flvcr1a*^fl/fl^;*Cdh5-Cre*^ERT2^ tumors. **M** Confocal images of CD31 (green) and NG2 (red) stained thick tumor Sects. (20 µm) from *Flvcr1a*^fl/fl^ and *Flvcr1a*^fl/fl^;*Cdh5-Cre*^ERT2^ mice. Scale bar: 50 µm. **N** Quantification of pericyte coverage as % NG2 area/CD31 area. *LLCs* Lewis Lung Carcinoma cells, *TAM* tamoxifen. Data are representative of at least 3 independent experiments and are expressed as mean ± SEM. *p < 0.05; ***p < 0.001; ****p < 0.0001. For statistical analyses, an unpaired Student’s t test was used

**Fig. 6:**
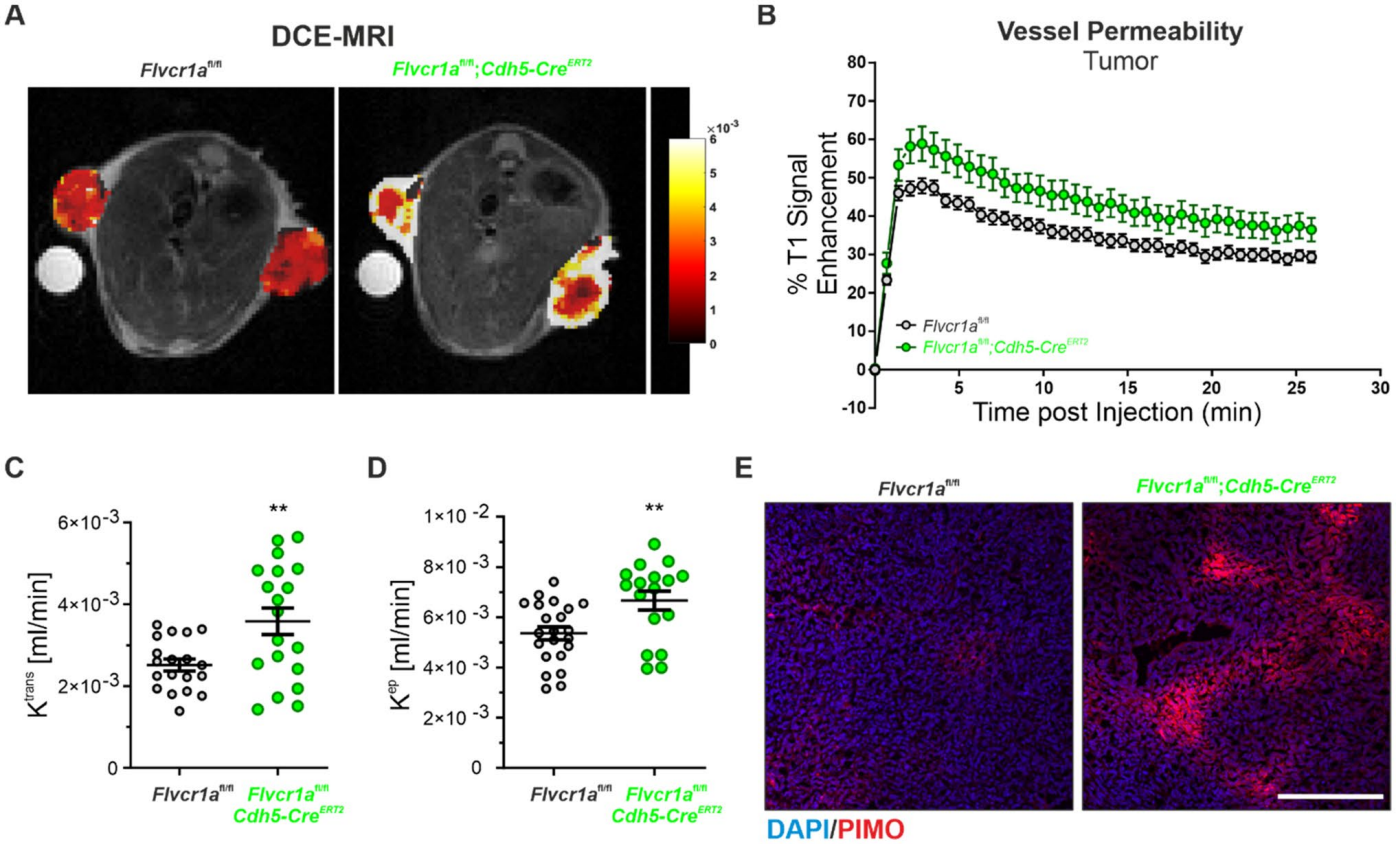
Endothelial *Flvcr1a* targeting increases tumor vessel permeability and hypoxia. **A**–**D** DCE-MRI analysis to evaluate tumor vessel permeability in LLC xenografts developed in *Flvcr1a*^fl/fl^;*Cdh5-Cre*^ERT2^ and *Flvcr1a*^fl/fl^ mice. The **B** enhancement curve, **C** K^trans^ constant, and **D** k^ep^ constant are shown. **E** Immunofluorescence analysis on *Flvcr1a*^fl/fl^ and *Flvcr1a*^fl/fl^;*Cdh5-Cre*^ERT2^ tumor sections stained with hypoxia marker pimonidazole (red) and DAPI (blu). Scale bar: 200 µm.; *ECs* endothelial cells, *PIMO* pimonidazole, *DCE-MRI* Dynamic Contrast-Enhanced Magnetic Resonance Imaging. Data are representative of at least 3 independent experiments and are expressed as mean ± SEM. **p < 0.01; For statistical analyses, an unpaired Student’s t test was used

To assess the role of FLVCR1a in adult neo-angiogenesis, the vasculature of tumors grown in *Flvcr1a*;*Cdh5-Cre*^ERT2^ mice was analyzed. To this purpose, Lewis lung carcinoma (LLC) cells were subcutaneously (s.c.) implanted in tamoxifen-treated *Flvcr1a*;*Cdh5-Cre*^ERT2^ mice (Fig. 5A). LLC xenografts were dissected after 14 days when tumor burden was mostly comparable between Flvcr1a;Cdh5-CreERT2 and control mice (Fig. S2), and the tumor vasculature was analyzed on histological sections following staining with the endothelial cell marker CD31 (Fig. 5B). Importantly, the total vascular area was significantly increased in *Flvcr1a*;*Cdh5-Cre*^ERT2^ tumors (Fig. 5C). Nevertheless, *Flvcr1a*;*Cdh5-Cre*^ERT2^ and control mice had comparable tumor vessel density, as indicated by the quantification of vessels number and of the vascular volume of perfused vessels measured by DCE-MRI (Fig. 5D and E, respectively). Conversely, *Flvcr1a;Cdh5-Cre*^ERT2^ tumor blood vessels displayed a significantly larger average size compared to blood vessel in control tumors (Fig. 5F), thus explaining the increase in the vascular area (Fig. 5C). In particular, vessels distribution was different in *Flvcr1a*;*Cdh5-Cre*^ERT2^ tumor, with a lower number of small capillaries and a higher number of larger ones (Fig. 5G). To obtain a quantitative evaluation of the spatial organization of tumor blood vessels, CD31 staining was performed on thick tumor sections, and microscopy images were processed to infer some vascular descriptive parameters as vessels perimeter and Feret’s diameter (Fig. 5H). In accordance with the presence of enlarged blood vessels, the average vessel perimeter was increased in *Flvcr1a*;*Cdh5-Cre*^ERT2^ tumor (Fig. 5I). Furthermore, the vascular architecture in tumors of *Flvcr1a*;*Cdh5-Cre*^ERT2^ mice was more irregular, tortuous, and disorganized (Fig. 5J). To elucidate whether the presence of larger vessels might be related to an increased EC proliferation, the number of EC nuclei per vessel perimeter was measured (Fig. 5K). However, the number of ECs per vessel was even lower than in control vessels (Fig. 5L), thus suggesting that increased vessel size was not associated to increased EC proliferation but rather to a reduced integrity and maturation. To address this point, IF staining for the pericytes’ marker NG2 was performed on tumor sections to investigate if FLVCR1a could affect the process of pericytes recruitment during vessel maturation (Fig. 5M). This analysis revealed a mild but significant reduction of direct interaction between pericytes and TECs in LLC tumors of *Flvcr1a*;*Cdh5-Cre*^ERT2^ mice (Fig. 5N). To investigate the vascular function in *Flvcr1a*;*Cdh5-Cre*^ERT2^ tumors, tumor vessel permeability was measured by DCE-MRI (Fig. 6A–D). Notably, tumor vessel permeability was significantly enhanced in *Flvcr1a*;*Cdh5-Cre*^ERT2^ mice compared to controls, as indicated by the quantitative analysis of k^trans^ and k^ep^ parameters (Fig. 6C–D). Alterations in vessel permeability can lead to reduced perfusion. To address this point, tumor oxygenation was evaluated in *Flvcr1a*;*Cdh5-Cre*^ERT2^ tumors. Staining for the hypoxia marker pimonidazole revealed increased hypoxic LLC tumor area in *Flvcr1a*;*Cdh5-Cre*^ERT2^ mice (Fig. 6E). Taken together, these results highlight the pivotal role of FLVCR1a in establishing proper vascular organization and ensuring vessel integrity in adult neo-angiogenesis.

### FLVCR1a is required for human pathological angiogenesis in vitro

**Fig. 7:**
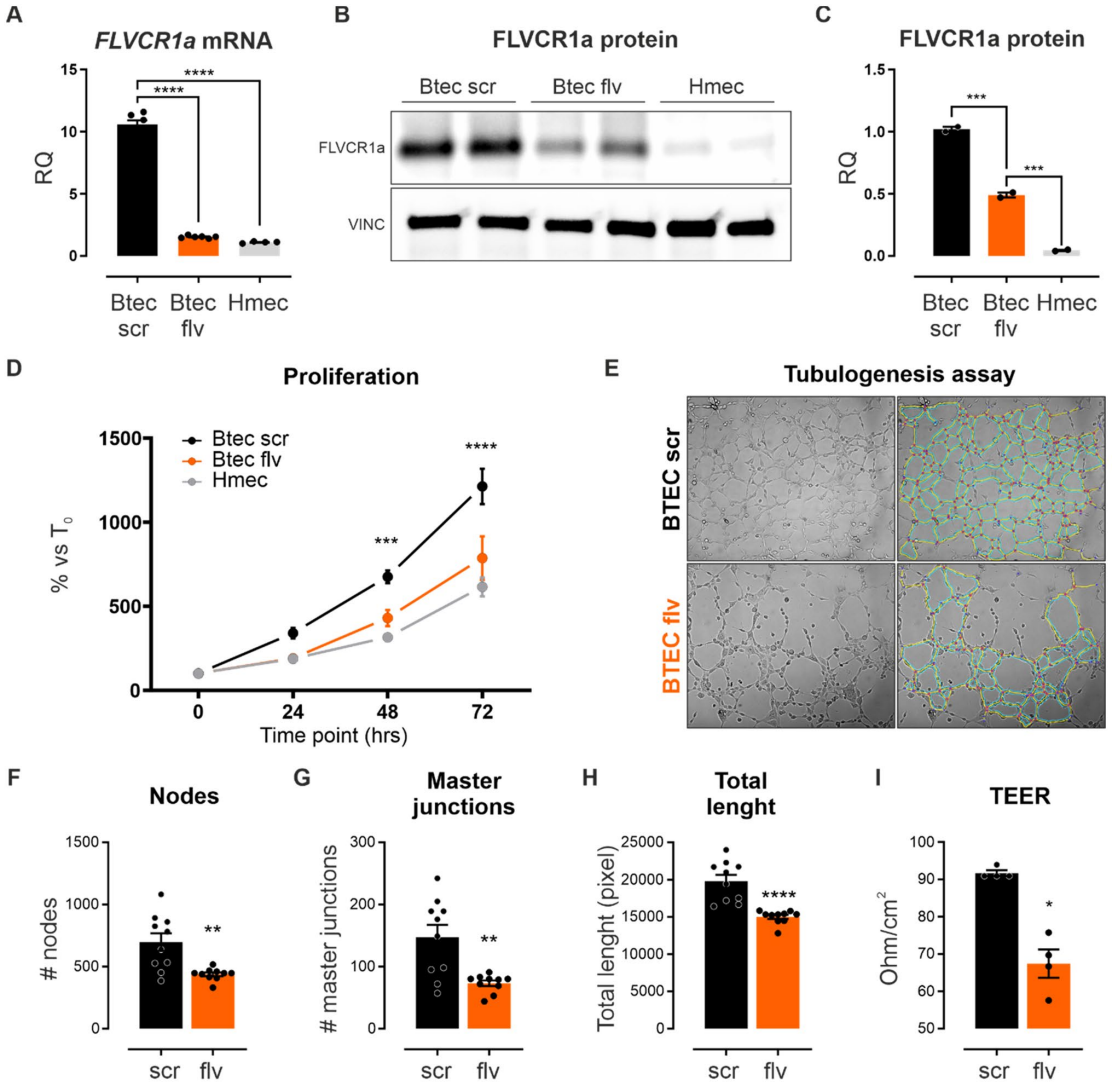
FLVCR1a modulates the angiogenic potential of human tumor-derived ECs in vitro. **A** qRT-PCR analysis showing *FLVCR1a* mRNA levels in normal (Hmec) and tumor-associated (Btec) human ECs. **B** Western Blot analysis showing FLVCR1a protein expression in Btec and Hmec. The protein quantification is shown in **C**. Proliferation of control Btec (scr), *FLVCR1a*-silenced Btec (flv), and Hmec at multiple time points. **E** in vitro tubulogenesis assay performed with control (scr) and *FLVCR1a*-silenced (flv) Btec. **F**–**H** Quantification of **F** number of nodes, number of **G** master junctions, and **H** total length of the networks obtained with Btec-scr and Btec-flv. **I** Transendothelial resistance (TEER) measurement performed on control (scr) and *FLVCR1a*-silenced (flv) Btec. Data are representative of at least 3 independent experiments and are expressed as mean ± SEM. *p < 0.05; **p < 0.01; ***p < 0.001; ****p < 0.0001. For statistical analyses, an unpaired Student’s t test was used

To confirm the relevance of FLVCR1a in human aECs, FLVCR1a expression levels were assessed in breast tumor-derived human endothelial cells (BTECs) as well as in microvascular ECs isolated from a healthy adult tissue, i.e., the human dermis (HMECs). Consistent with previous data on human ECs (Fig. 1G), FLVCR1a expression was much higher in BTECs, as compared to HMECs (Fig. 7A–C). To elucidate the role of FLVCR1a in human TECs, BTECs were infected with a lentiviral vector carrying the short hairpin RNA (shRNA) specific for human FLVCR1a (BTEC-flv) or a “scramble” shRNA as control (BTEC-scr). FLVCR1a transcript and protein levels were significantly reduced in *FLVCR1a*-silenced BTECs by approximately 90% and 80%, respectively (Fig. 7A–C). To investigate how FLVCR1a affects human ECs behavior, cell proliferation was evaluated in non-tumoral HMECs and tumor-derived BTECs (with or without FLVCR1a) by performing cell count at different time points. Firstly, BTECs displayed a higher proliferative rate compared to non-tumoral HMECs (Fig. 7D). Moreover, *FLVCR1a* downmodulation was sufficient to reduce BTEC proliferative rate, bringing it down to a level comparable to HMECs (Fig. 7D). Taken together, these findings suggest that there is a correlation between FLVCR1a expression and EC proliferative capacity. To investigate how the reduced EC proliferation might affect the formation of a vascular network, an in vitro tubulogenesis assay was performed with *FLVCR1a*-silenced and control BTECs (Fig. 7E). The complexity of the interconnections inside the network as well as the capillary network extension in space were quantified in terms of number of nodes, number of master junctions, and total length of the newly formed “tree” (Fig. 7F–H). The capillary network formed by *FLVCR1a*-silenced BTECs showed an altered spatial organization, thus suggesting that FLVCR1a is required to form vascular networks not only in developmental angiogenesis but also in tumors. To assess the impact of FLVCR1a loss on endothelial cell functionality, trans-endothelial resistance (TEER) was measured in BTECs upon FLVCR1a silencing to evaluate the permeability of the endothelial cell monolayer. Importantly, FLVCR1a-silenced BTECs displayed a reduced TEER thus indicating enhanced permeability (Fig. 7I). These results suggest that FLVCR1a is involved in the control of endothelial barrier functions in human TECs.

These data, taken together, indicate that FLVCR1a has a crucial role in human TECs by controlling their ability to proliferate and form a functional and properly organized capillary network in vitro.

## Discussion

FLVCR1a is a cell membrane heme exporter from the cytosol toward the extracellular space [13, 18]. The generation of the endothelial-specific constitutive knockout mouse demonstrates an EC autonomous function for FLVCR1a in developmental angiogenesis. In particular, endothelial *Flvcr1a* null embryos develop the primitive vascular network via vasculogenesis but display defective angiogenesis, resulting in extensive hemorrhages, skeletal malformations, and embryonic lethality between E15.5 and 16.5 [12, 19, 20].

**Fig. 8:**
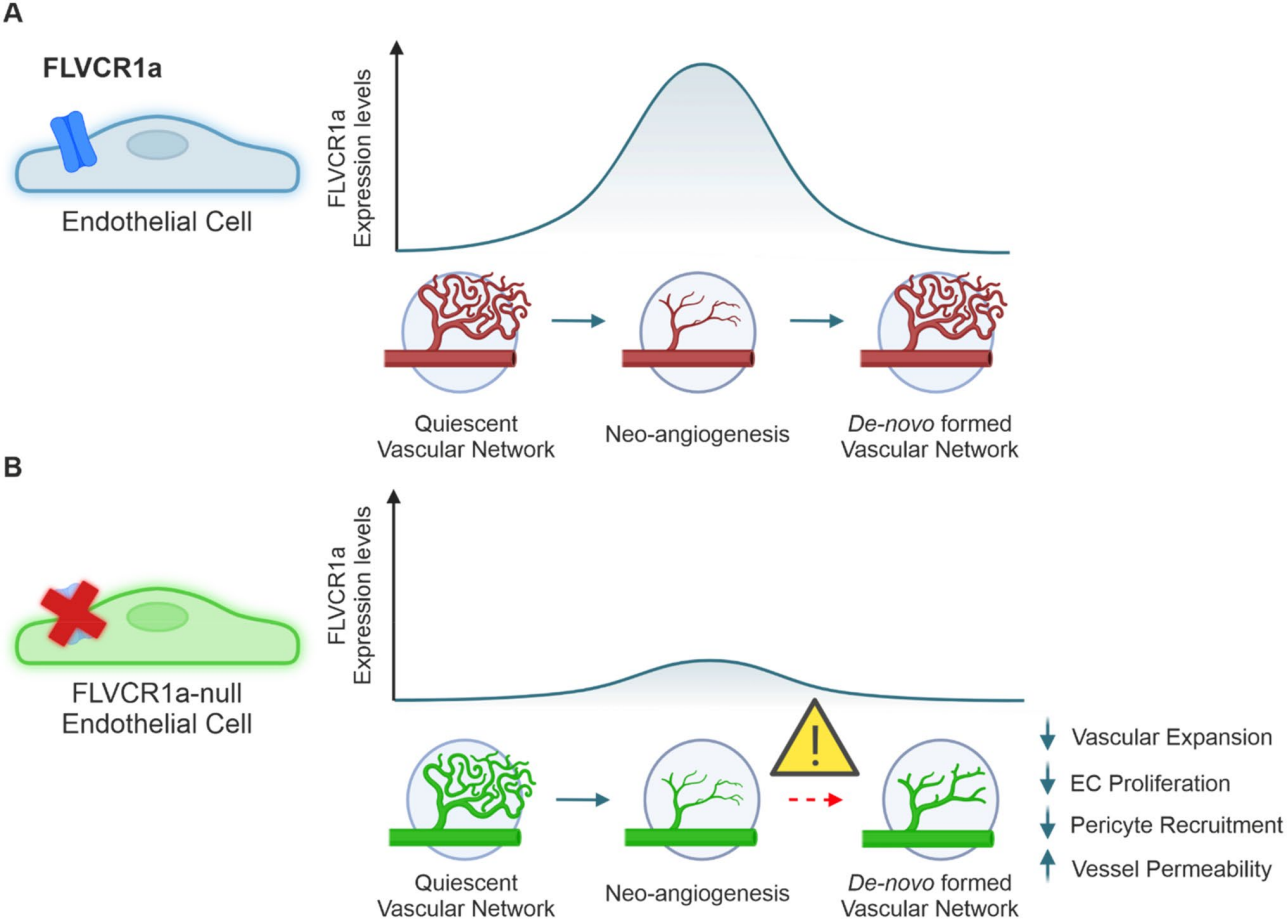
Endothelial FLVCR1a is required for proper angiogenesis. **A** Schematic representation of endothelial FLVCR1a expression during angiogenic vascular growth. FLVCR1a is low in the quiescent vasculature and strongly increases in aECs that have to proliferate and arrange in a new vascular network. Once the de novo vasculature is fully formed and mature, FLVCR1a levels decrease. **B** Schematic representation of vascular defects upon endothelial Flvcr1a targeting. Given the importance of FLVCR1a in aECs, FLVCR1a deficiency does not affect fully formed vascular networks. Conversely, endothelial-specific FLVCR1a targeting severely compromises neo-angiogenesis by reducing vascular expansion, ECs proliferation, and pericyte recruitment while increasing vessel permeability

We found FLVCR1a to be highly expressed in aECs involved in the formation of a new vascular network, whereas its expression level progressively declines once the vascular network is fully formed (Fig. 8). Consistent with the essential role in aECs, endothelial FLVCR1a targeting profoundly impairs the formation of a fine and well-structured vascular tree in multiple models of developmental angiogenesis. Firstly, endothelial *Flvcr1a* null embryos show a compromised microvessels architecture that leads to massive hemorrhages [12]. Similarly, post-natal endothelial *Flvcr1a*-targeting causes evident vascular defects in the developing P8 mouse retina with reduced vascular outgrowth and defective organization of the retinal vascular network. These results agree with previous works showing a reduced ability of *Flvcr1a*-deficient microvascular ECs from human dermis to form a well-patterned capillary network in vitro [12]. These vascular defects are associated to a decrease in ECs proliferation in the developing retina. Notably, the critical role of FLVCR1a in developmental angiogenesis is evolutionary conserved, as illustrated in zebrafish morphants, which display defective spatial organization of intersegmental vessels associated to a hemorrhagic phenotype [15].

Aberrant angiogenesis is a critical step associated to tumor development. Noteworthy, *Flvcr1a* levels are even higher in ECs isolated from LLC xenografts as compared to embryonic aECs, indicating a crucial role of the cell membrane transporter also in the tumor context. Furthermore, *Flvcr1a* expression in LLC-derived TECs is very much higher when compared to qECs derived from the healthy adult lung of the same mouse, further suggesting that its activity is specifically required in aECs. Notably, similar results were also found in ECs isolated from human lung cancer patients when comparing *FLVCR1a* single-cell expression data between tumor-associated ECs and ECs of the peri-tumoral healthy tissue. These data agree with previous findings showing an increased *FLVCR1a* expression in human endothelial cell lines derived from tumors or healthy tissues, respectively [21]. Consistent with the essential role of FLVCR1a in tumor aECs, LLC tumor vasculature lacking FLVCR1a is characterized by an increased number of enlarged vessels that fail to properly organize in a mature network and exhibit high tortuosity and enhanced permeability. These findings agree with those observed in *Flvcr1a*-silenced human ECs derived from breast cancer (BTECs) which show a reduced ability to organize in space and display reduced trans-endothelial resistance (TEER) in vitro. In accordance with a defective vessel integrity, *Flvcr1a* null vessels display a reduced pericyte coverage both in the developing retina as well as in LLC xenografts. Notably, vessel structure and permeability are not altered in quiescent blood vessels of healthy tissues lacking *Flvcr1a*, further suggesting a restricted function of the transporter in aECs.

Collectively, these results highlight the importance of FLVCR1a in the formation of a well-structured and functional three-dimensional vascular network. The described vascular defects may be directly linked to the heme-related function of FLVCR1a. Heme is an iron-containing porphyrin involved in multiple biological processes, acting as a crucial cofactor required for electron transport, energy generation, and chemical transformation [18]. Accordingly, proliferating cells are highly dependent on heme synthesis and sensitive to heme homeostasis perturbations [15, 22, 23]. Recent studies showed the pivotal role of heme in ECs. Indeed, endothelial heme deficiency due to the genetic targeting of phosphoglycerate dehydrogenase (PHGDH), the enzyme required for the synthesis of the heme precursor glycine, reduces EC proliferation and survival thus compromising vessel formation [23]. Notably, heme-analog supplementation rescues the angiogenic defects caused by endothelial PHGDH targeting, thus supporting a direct involvement of heme in angiogenesis. Noteworthy, *Phgdh* is overexpressed in TECs compared to healthy ECs, suggesting a driving role of heme also in tumor angiogenesis [23]. Additional works pointed out that the endothelial targeting of ferrochelatase (FECH), the last enzyme in heme biosynthesis, compromises angiogenesis both in vitro and in vivo. In particular, FECH targeting reduces human retinal ECs (HRECs) proliferation and migration, thus impairing their ability to correctly form tubule-like structures in vitro [24]. In agreement with these observations, inactivation of the heme cellular exporter FLVCR1a, with an established role as a positive regulator of heme synthesis [14], results in similar vascular defects. Interestingly, endothelial FECH is upregulated in experimental models of human ocular neovascularization diseases as neovascular age-related macular degeneration (AMD) and retinopathy of prematurity (ROP) [24, 25]. Inhibition of FECH ameliorates the disease in both models [24, 25], indicating that heme synthesis targeting may be a promising strategy to counteract aberrant neovascularization in several sight-threatening disorders.

The functional link between FLVCR1a expression and cell proliferation was demonstrated in multiple cell types, including committed erythroid progenitors in mice and zebrafish [15], intestinal epithelial cells [26]. Consistent with a fundamental role of heme in highly proliferating cells, *Flvcr1a* is overexpressed in several tumor types, including bovine papillomavirus-associated urinary bladder cancer, colorectal cancer, hepatocellular carcinoma, lung cancer, synovial sarcoma, and prostate cancer [14, 22, 27, 28]. The results described here pointed out a pivotal role of FLVCR1a also for proliferation of aECs. Indeed, endothelial *Flvcr1a* deficiency reduces EC proliferation in the angiogenic vasculature of P8 mouse retina. These data agree with previous evidence showing that *Flvcr1a*-downmodulation reduces proliferation of HMECs [12]. The link between Flvcr1a expression and EC proliferation is further supported by the observation that *Flvcr1a* expression in mouse embryonic ECs undergoes a drastic drop off at E13.5, which coincides with the transition from proliferating angiogenic endothelium to a more quiescent endothelium. Remarkably, FLVCR1a is strongly upregulated in angiogenic TECs, which are highly proliferating ECs compared to adult quiescent ECs [21]. In line with this, *Flvcr1a*-silencing in BTECs reduces cell proliferation to levels comparable to healthy HMECs. Collectively, these results strongly support the importance of FLVCR1a in EC proliferation. Moreover, this suggests that vascular defects upon endothelial *Flvcr1a*-targeting may be at least in part due to reduced EC proliferation.

To start angiogenesis, ECs undergo a metabolic rewiring aimed to sustain increased cell proliferation and migration [29]. Recent evidence pointed out that angiogenesis requires a fine coordination of both glycolysis and mitochondrial metabolism [11, 30, 31]. On the one hand, glycolysis shutdown by blocking the glycolytic activator PFKFB3 leads to tumor vessels normalization and enhanced drug delivery. On the other hand, loss of respiratory chain complex III in ECs impairs retinal and tumor angiogenesis [30]. Similarly, the benzoquinone embelin mitochondrial uncoupler impairs neo-angiogenesis during tumor growth and wound healing [32]. In particular, embelin-treated tumor vessels show increased average vessel diameter, with a preferential regression of small capillaries, increased vessel leakage, and increased tumor hypoxia [32]. Interestingly, embelin administration specifically targets proliferating ECs and not quiescent ECs, supporting the importance of mitochondrial respiration in angiogenic ECs [32]. Heme metabolism plays a pivotal role in the control of metabolic adaptations in highly proliferating cells, including cancer cells and aECs [14, 22]. In particular, alterations in heme homeostasis strongly perturb mitochondrial metabolism [33]. Endothelial heme deficiency upon PHGDH genetic targeting causes mitochondrial defects resulting in reduced EC survival and defective angiogenesis [23]. Moreover, FECH loss in retinal ECs significantly affects mitochondria homeostasis by increasing fragmentation and reducing mitochondrial membrane potential, thus impairing the angiogenic properties of ECs [33, 34]. Furthermore, *Flvcr1a*-deficient TECs display increased oxidative phosphorylation (OXPHOS) and ATP synthesis, resulting in reduced cell proliferation [14]. Finally, *Flvcr1a*-deficient ECs display heavily damaged mitochondria characterized by an aberrant morphology [12]. In this view, the metabolic rewiring associated to *Flvcr1a*-targeting in ECs may be involved in the vascular defects described here. *Flvcr1a* targeting in ECs also affects metabolic pathways interlinked with TCA cycle [14, 21]. Moreover, heme synthesis is itself a cataplerotic pathway of the TCA cycle by consuming succinyl-CoA. Since TCA cycle intermediates are essential for biomass production in ECs [31], alterations in the availability of these molecules might contribute to the proliferative and angiogenic defects found in *Flvcr1a*-deficient vasculature.

Another unexplored possibility is that FLVCR1a may accomplish different functions unrelated to heme. Indeed, FLVCR1a belongs to the major facilitator superfamily (MSF) of transporters, whose members are specialized in the transport of small solutes across cell membranes in response to chemiosmotic gradients [35]. However, it remains unknown whether FLVCR1a might associate heme transport to the import/export of some ion or small molecule [36, 37]. Notably, ion trafficking (i.e., Ca^2+^, K^+^, Na^+^) and balance is critically involved in several steps of angiogenesis [29, 38, 39]. In this view, some phenotypes related to FLVCR1a deficiency in ECs might be partly due to a disbalance in ion exchange across the plasma membrane.

Finally, FLVCR1a, as many other plasma membrane proteins, may act as scaffold or modulator for a multitude of signaling pathways involved in angiogenesis. Among these pathways, Notch receptor is a major regulator of angiogenesis by regulating the acquisition of specialized tip/stalk phenotypes in aECs [40, 41]. In particular, Dll4–Notch1 signaling restricts tip-cell formation in response to VEGF, thereby establishing the adequate ratio between tip and stalk cells required for correct sprouting and branching patterns. Interestingly, alterations in Notch signaling leads to vascular defects in the developing retina and in zebrafish embryos which partly resemble Flvcr1a deficiency [42]. Moreover, the *Notch*-deficient tumor blood vessels are enlarged, tortuous and less mature, and promote tumor hypoxia [43], again resembling some features of endothelial *Flvcr1a*-deficient tumors. These observations lay the basis for future studies aimed at understanding whether there was any connection between Flvcr1a and Notch pathways.

Collectively, data arising from this study deeply elucidate the relevance of FLVCR1a in angiogenic endothelium. These findings lay the basis for future studies aimed at developing new therapeutic strategies to target angiogenic ECs in human diseases characterized by aberrant neovascularization.

## Methods

### Cell cultures

Human adult dermal microvascular endothelial cells (HMECs) were purchased by Lonza, propagated in EndoGRO-MV-VEGF Complete Culture Media Kit (SCME003 Merck Millipore) and used up to passage 12. Breast tumor-derived endothelial cells (BTECs) from human breast lobular-infiltrating carcinoma biopsy were isolated and characterized in the laboratory of Professor Benedetta Bussolati, Department of Molecular Biotechnology and Health Sciences, University of Torino, Italy [44, 45]. BTECs were maintained in EndoGRO-MV-VEGF Complete Culture Media Kit (SCME003 Merck Millipore). Lewis lung carcinoma LL/2 (LLC1) cells (ATCC: CRL-1642) were cultured in Dulbecco’s modified Eagle’s medium (DMEM, high glucose, GlutaMAX supplement; Gibco by Thermo Fisher Scientific, Waltham, MA USA, catalog n° 61965059) supplemented with 10% heat-inactivated low-endotoxin fetal bovine serum (FBS; Gibco by Thermo Fisher Scientific, Waltham, MA USA, catalog n° 10270106). All cell media were ordinarily supplemented with antibiotics (100 U/ml penicillin and 100 µg/ml streptomycin; Gibco by Thermo Fisher Scientific, Waltham, MA USA, catalog n°15140122). Cells were maintained in a 37 °C and 5% CO2 air incubator and routinely screened for the absence of mycoplasma contamination.

### *FLVCR1a* gene silencing

*FLVCR1a* silencing in BTECs was performed as described in [12]. Briefly, a shRNA targeting the sequence correspondent to the first exon of the human *FLVCR1* gene was used to specifically down-regulate *FLVCR1a* (TRC Lentiviral pLKO.1 Human FLVCR1 shRNA set RHS4533-EG28982, clone TRCN0000059599; Dharmacon, Lafayette, CO, USA). For control cells, a pLKO.1 lentiviral vector expressing a scramble (scr) shRNA was used. The lentiviruses were produced in HEK293FT cells. Cells were infected with the lentiviruses in the presence of Sequabrene™ (S2667 Sigma-Aldrich, St. Louis, MO USA). Following lentiviral transduction, cells were maintained in selective medium containing 0.002 mg/ml puromycin (Puromycin dihydrochloride from Streptomyces alboniger, Sigma-Aldrich, St. Louis, MO USA, catalog n° P8833).

### Cell proliferation

To test the in vitro proliferative potential, 1 × 10^4^ cells were plated on a 12 multi-well plate, and every 24 h cell were trypsinized, stained with methylene blue, and counted by using Countess Automated Cell Counter (Thermo Scientific).

## Western blot analysis

To assess FLVCR1a expression, BTECs were lysed by rotation for 30 min at 4 °C in RIPA buffer (150 mM NaCl, 50 mM Tris–HCl pH 7.5, 1% Triton X-100, 0.5% Sodium deoxycholate, 0.1% SDS, 1 mM EDTA). The buffer was freshly supplemented with 1 mM phosphatase inhibitor cocktail (Sigma Aldrich, St. Louis, MO USA, catalog n° P0044), 1 mM PMSF (Sigma Aldrich, St. Louis, MO USA, catalog n° 93482-50ML-F), and protease inhibitor cocktail (La Roche, Basel, CH, catalog n° 04693116001). The cell lysate was clarified by centrifugation for 10 min at 4 °C. Protein concentration in the supernatant was assessed by Bradford assay. For FLVCR1a protein detection, 10 ug of protein extracts was incubated 10 min at 37 °C with 1 uL of PNGase-F from Elizabethkingia meningoseptica (Sigma Aldrich, St. Louis, MO USA, catalog n° P-7367) to remove protein glycosylation. Before loading on 4–15% mini-PROTEAN TGX precast gel (Bio-Rad, Hercules, CA USA, catalog n°4568084), samples were incubated 5 min at 37 °C (FLVCR1) or 10 min at 95 °C (Vinculin) in 4 × laemmli buffer freshly supplemented with 8% 2-mercaptoethanol. The primary antibodies and dilutions are as follows: FLVCR1 (C-4) (Santa Cruz Biotechnology, Dallas, TX USA, catalog n° sc-390100; 1:500); Vinculin (home-made, 1:8000); Anti-myc tag antibody (1:1000 ab9106 Abcam). The revelation was assessed using the ChemiDoc Imaging System (Bio-Rad, Hercules, CA USA).

### Tubulogenesis assay

In vitro formation of capillary-like structures of BTECs was studied on growth factor-reduced Matrigel (BD Bioscience, Franklin Lakes, NJ, USA) in 24-well plates. Cells (3.5 × 10^4^ cells/well) were seeded onto Matrigel-coating in EndoGRO MV-VEGF (Millipore, Merck, Italy). Cell organization onto Matrigel was observed with a Nikon Eclipse Ti E microscope using a Nikon Plan 10 ×/0.10 objective and cells were kept at 37 °C and 5% CO_2_ during the experiment. Images were acquired after 8 h. At least three independent experiments were done for each experimental condition. ImageJ’s Angiogenesis Analyzer was used to analyze the number of nodes and master junctions as well as total length.

## Transendothelial resistance (TEER)

Transendothelial resistance of BTECs was measured by using Volt/Ohm (TEER) Meter EVOM2 (WPI). Briefly, 10 × 10^4^ cells/well BTEC were seeded on 0.4 µm pore size 24-well-Transwell® inserts (Corning), and after 2 week, the cell monolayer’s TEER was measured through the Cell Culture Cup Chambers “ENDOHM-6G” of EVOM2.

## Bioinformatic analysis

The human single-cell gene expression data generated by Goveia et al. were obtained from the lung Tumor EcTAX site (https://www.vibcancer.be/software-tools/lungTumor_ECTax), together with the cluster label of each cell. Endothelial cells belonging to the clusters "tip cell," "immature," "capillary alveolar type I," "capillary alveolar type II," “capillary activated,” “capillary intermediate,” and “capillary scavenging” were retained. The FLVCR1 expression level in each cell was expressed as the difference with the expression of the individual cell with the lowest FLVCR1 expression and then log_10 transformed. The Mann–Whitney test was used to assess the significance of the difference in expression among clusters (capillary ECs vs angiogenic stalk/tip ECs).

### Zebrafish models

Zebrafish embryos were raised and maintained according to standard procedures at 28.5 °C as previously described [46]. Embryos were staged using hours post fertilization (hpf) and standard morphologic criteria [47]. Transgenic kdrl:GFPs843 zebrafish expressing green fluorescent protein (GFP) under the control of the kdrl promoter were used to study vascular development [48]. To visualize GFP fluorescence by whole-mount microscopy, 0.003% phenylthiourea (PTU, Sigma) was added to egg water at 24 hpf to prevent pigment formation in embryos. Oligomorpholinos (MO) were designed (GeneTool® oligo design) either to block splicing at specific exon–intron junctions (MoI1Ex2), and consequently lead to aberrant transcripts and frame-shifted translation, or to anneal to the ATG start codon (MoATG) and inhibit translation initiation. Zygotes were collected at one-cell stage and injected with 4 ng of MO, in the presence of phenol red for subsequent selection. MO sequences were as follows: Control, 5′-CCTCTTACCTCAGTTACAATTTATA-3′; MoATG, 5′-CCTGGAGAAACTCACCTGCCACCAT-3′; MoIn1Ex2, 5′-ACCAAGCTGACGGGAAATAAAGAGA-3′. For rescue experiments, murine *Flvcr1a* cDNAs were cloned into the pCS2 + expression vector and cRNA was synthetised using RNA transcription kit (Ambion, Austin, TX) and co-injected with MOs in zygotes at one-cell stage. MoATG sequence did not match *Flvcr1a* cRNA. Experimental procedures related to fish manipulation followed previously reported recommendations and conformed with the Italian regulations for protecting animals used in research, including DL 116/92. The Ethics committee of the University of Torino approved this study. Larvae were anesthetized and then sacrificed by ice chilling.

### Mouse models

Constitutive endothelial-specific *Flvcr1a*-knockout mice were generated by crossing *Flvcr1a*^fl/+^; *Tie2*-Cre male mice with *Flvcr1a* female mice. Mice were genotyped by polymerase chain reaction (PCR) using DNA from yolk sacs biopsies. The primers used for genotyping are listed below. Embryos were obtained from timed pregnant females with the morning of vaginal plug considered E0.5.

Inducible endothelial-specific *Flvcr1a*-null mice (*Flvcr1a*;*Cdh5-Cre*^ERT2^) were generated by crossing *Flvcr1a* mice with *Cdh5*-*Cre*^ERT2^ mice (Tg(*Cdh5-Cre*/^ERT2^)1Rha, kindly provided by Ralf H. Adams) on a C57BL/6 background. Mice were genotyped by PCR on genomic DNA from tail biopsies using primers listed below. To induce *Flvcr1a* gene deletion in the developing retinal vasculature, *Flvcr1a*;*Cdh5-Cre*^ERT2^ pups were injected with 50ul of 2 mg/mL tamoxifen solution tamoxifen (Sigma Aldrich St. Louis, MO USA, catalog n°T5648) into the stomach for three consecutive days, from post-natal day (P) 1 to P3. Eyes were dissected at P8 to analyze angiogenesis. To inactivate *Flvcr1a* selectively in ECs in the adult mouse, 4–6-week-old *Flvcr1a*;*Cdh5-Cre*^ERT2^ mice were treated intraperitoneally with 1 mg/day tamoxifen (Sigma Aldrich St. Louis, MO USA, catalog n°T5648) for 5 consecutive days, followed by 1 additional day after a 2-days treatment free interval. Tamoxifen-treated *Flvcr1a* mice were used as controls. For all the experiments with the inducible knockout model, tamoxifen solution was prepared as previously described [49]. All the mice were provided with food and water ad libitum. All experiments with animals were approved by the Italian Ministry of Health.

The *FLVCR1-myc* line was generated by CRISPR/Cas9, inserting the GAACAAAAACTCATCTCAGAAGAGGATCTG MYC sequence in frame at the 3′ end of the *FLVCR1* coding region. For this, a sgRNA targeting the sequence AATCTTCACTCTGAAGAAGC was generated by in vitro transcription. Briefly, oligonucleotides AGGGAATCTTCACTCTGAAGAAGC and AAACGCTTCTTCAGAGTGAAGATT were annealed and cloned into the BssI sites of plasmid pgRNA-basic [50]. The sgRNA was transcribed from the resulting plasmid with the MEGAshortscript T7 Kit (Life Technologies) and purified with the MEGAclear Kit (Life Technologies). Cas9 mRNA was produced by in vitro transcription from the pT7-Cas9 plasmid [50] using the mMESSAGE mMACHINE T7 Ultra Kit (Life Technologies) and purified with the MEGAclear Kit. The purified gRNA was microinjected into fertilized FVB/N mouse oocytes together with the Cas9 mRNA and the synthetic ssDNA 5′-AGTTGATAGTCGGGTAGATCCAAAACCCAAAGTGATGGTGTCTATACAGTCGGAATCTTCACTCGAACAAAAACTCATCTCAGAAGAGGATCTGTGAAGCCGGTGTGGCTGCCCCTCAACATGGGCATGTGGCTTCGTCTTTGGGCAGCTGTGTGAGGTG-3′. The edited alleles were confirmed by direct sequencing of the target region. Genotyping of embryos was performed by PCR. The primers used for genotyping are listed below. Mice used at Instituto Gulbenkian de Ciência (IGC) were bred and maintained under specific pathogen-free (SPF) conditions. Protocols were approved in a two-step procedure, by the Animal Welfare Body of the IGC and by the Portuguese National Entity that regulates the use of laboratory animals in research (DGAV—Direção Geral de Alimentação e Veterinária). Experiments on mice followed the Portuguese (Decreto-Lei nº 113/2013) and European (Directive 2010/63/EU) legislation, concerning housing, husbandry, and animal welfare. C57BL/6J wild-type mice were obtained directly from the IGC animal facility.

**Table:** 

Target	Forward primer (5′–3′)	Reverse primer (5′–3′)	Band length (base pairs)
*Flvcr1a* null allele	TCTAAGGCCCAGTAGGACCC	AGAGGGCAACCTCGGTGTCC	320
*Flvcr1a* wild-type allele	TCTAAGGCCCAGTAGGACCC	GAAAGCATTTCCGTCCGCCC	242
*Flvcr1a* floxed allele	TCTAAGGCCCAGTAGGACCC	GAAAGCATTTCCGTCCGCCC	280
*Tie2-Cre transgene*	GGACATGTTCAGGGATCGCCAGGCG	GCATAACCAGTGAAACAGCATTGCT	269
*Cdh5-Cre transgene*	ACACCTGCTACCATATCATCCTAC	CATCGACCGGTAATGCAG	330

### Retina dissection and immunostaining

Eyes were fixed for 2 h (hrs) in paraformaldehyde (PFA) 4% at 4 °C. Retina dissection was performed at post-natal day 8 as previously described [49]. After retina dissection, 4 to 5 radial incisions were done using spring scissors to create a 'petal' shape. The retinas were put in cold (− 20 °C) methanol for at least 20 min (until turning white) before proceeding with immunostaining. Next, the retinas were washed in Phosphate-buffered saline 1 × (PBS 1 ×) and then covered with 100 μl of permeabilization/blocking solution (PBS + 0.3% Triton + 0.2% BSA) + 5% BSA on gentle shake for 1 h at room temperature (RT). Next, the retinas were incubated with 100 μl of selected primary antibodies overnight at 4 °C with gentle shaking. Next, the retinas were wash retinas 4 × 10 min in PBS + 0.3% Triton (PBSTX) and incubated with the appropriate fluorescent secondary antibody (diluted 1/200 in PBSTX) overnight at 4 °C. Finally, retinas were washed in PBS and mounted on a cover glass.

### Xenograft tumor model

For the LLC xenograft model, 5 × 10^5^ LL/2 (LLC1) murine cells suspended in 100 µl PBS were injected subcutaneously into the flanks of immunocompetent syngeneic C57BL/6 mice. For tumor induction in *Flvcr1a*;*Cdh5-Cre*^ERT2^ mice and controls, 4–6 week-old mice were treated intraperitoneally with tamoxifen (Sigma Aldrich St. Louis, MO USA; 1 mg/day for 5 consecutive days and 1 additional day after a 2-days treatment free interval) one week before LLC cells injection.

### Magnetic resonance imaging for tumor volume measurement

The tumor volume was measured by Magnetic Resonance Imaging (MRI) by using a 7.1 T Bruker Avance 300 scanner. Before undergoing MRI, animals were anesthetized by intramuscular injection of 5 mg/kg of xylazine (Rompun; Bayer) and 20 mg/kg of tiletamine/zolazepam (Zoletil 100; Virbac). The animals were then scanned at day 4, 6, 8, 10, 14, and 17 post inoculation through T2-weighted (T2w) images acquired with the following parameters: repetition time (TR) = 4000 ms; effective echo time (TE) = 55 ms; slice thickness = 0.60 mm; 16 slices; field of view (FOV) = 3.50 cm; matrix = 128 × 128; number of averages (NAV) = 6; total imaging time = 1 m 36 s). Tumor volume was assessed in each animal through MRI data analysis, carried out with FIJI Software. More in detail, the tumor area was delineated in each slice of T2w high-resolution images by manually drawing regions of interest along tumor borders. The tumor volume in each slice was estimated by multiplying each tumor area by the slice thickness (0.6 mm). Finally, the total tumor volume was estimated by adding up all the single slice volumes.

### Dynamic contrast-enhanced MRI (DCE MRI)

Dynamic Contrast-Enhanced (DCE) MRI was acquired on anesthetized mice with an Aspect M2 system (Aspect Magnet Technologies Ltd. Netanya, Israel) working at 1 T. Mice were anesthetized by injecting a mixture of 20 mg/kg tiletamine/zolazepam (Zoletil 100; Virbac, Milan, Italy) and 5 mg/kg xylazine (Rompun; Bayer, Milan, Italy) and placed supine in a solenoid Tx/Rx coil with an inner diameter of 3.5 cm. After the scout image acquisition, a T2w anatomical image was acquired with a Fast Spin Echo (FSE) sequence (TR 2500 s; TE 48 ms; number of slices 4; slice thickness 2.0 mm; FOV 40 mm; matrix 160 × 160; NAV 4; acquisition time 2 m 50 s). DCE–MRI was performed using an axial GRE SNAP sequence (TR 16 ms; TE 2.7 ms; flip angle 30°; number of slices 4; slice thickness 2.0 mm; slice gap 0.1 mm; FOV 40 mm; matrix 128 × 128; NAV 5; temporal resolution 42 s per image). The dynamic series was acquired with three initial pre-contrast T1w images and 37 dynamic T1w post-contrast images for a total examination time of 27 min. The contrast agent ProHance (Bracco Imaging, Milano, Italy) was injected intravenously at a dose of 0.2 mmol Gd/kg body weight. Before the contrast administration, a variable flip angle (VFA) map was acquired with the GRE SNAP sequence: NAV 5, 1 repetition, flip angles 5°–15°–30°–45°–60°–75°. All the DCE-MRI images were analyzed using in-house developed software (KMaps 0.1.4) [51]. ROIs were drawn on tumors and livers, and the % T1w enhancement over pre-contrast injection images was calculated along the acquired time points. Parametric maps of *K*^trans^ (the volume transfer constant between the intravascular and the extracellular extravascular space), *K*^ep^ (the rate constant from EES to blood plasma), and *V*_p_ (the plasmatic volume) were obtained on a voxel-by-voxel basis by applying an extended Tofts' model with an individually measured arterial input function (AIF) [52].

### Tumor hypoxia

Tumor hypoxia was analyzed by i.p. injection of pimonidazole hydrochloride (60 mg/kg) 1 h prior to dissection followed by immunostainings with RED 549 dye-conjugated anti-pimonidazole mouse IgG1 monoclonal antibody (RED 549-MAb1) (Hypoxyprobe™ RED 549 Kit).

### ECs isolation from LLC xenografts and adult lungs

Normal ECs and tumor-associated endothelial cells (TECs) were isolated from adult lungs and LLC subcutaneous tumors, respectively [11]. Briefly, tumors or lungs were dissected and minced into 1–2 mm fragments with a scalpel. Tissue pieces were incubated at 37 °C for 60 min in 10 ml of pre-warmed Dulbecco’s Phosphate Buffered Saline (DPBS) with Calcium and Magnesium (Lonza Pharma & Biotech, Basel, CH, catalog n BE17-513F) and 2 mg/ml Collagenase (Collagenase from Clostridium histolyticum, Type I, Sigma Aldrich St. Louis, MO USA, catalog n C0130), with regular shacking until a single-cell suspension was obtained. During this incubation, the cells were mechanically dissociated at 10 min intervals by pipetting. To stop the collagenase activity, DMEM (GIBCO by Thermo Fisher Scientific, Waltham, MA USA, catalog n 61965059) containing 10% FBS (GIBCO by Thermo Fisher Scientific, Waltham, MA USA, catalog n 1027106) was added to the cell suspension, gently pelleted, and rinsed with PBS. The cells in PBS were then filtered through a 40 mm Cell Strainer (Corning Life Sciences, Corning, NY USA, catalog n 352340). Single-cell suspension was centrifuged at 300×*g* for 10 min and ECs were isolated through MACS Technology by using nano-sized MicroBeads, following the manufacturer instructions. Particularly, a negative selection was performed using CD45 MicroBeads (Miltenyi Biotec, Bergisch Gladbach, DE, catalog n 130-052-301). CD45-negative cell fraction was then pelleted and incubated with CD31 MicroBeads (Miltenyi Biotec, Bergisch Gladbach, DE, catalog n 130-097-418) to isolate ECs (CD45−/CD31+ cell fraction).

### ECs isolation from mouse embryos

Embryos were isolated from wild-type mice at E9.5 or E13.5 and minced into 1–2 mm fragments with a scalpel. After rinsing in PBS, the tissue pieces were incubated for 45 min at 37 °C in 10 ml of pre-warmed PBS (phosphate buffered saline) with collagenase (0.5 mg/ml, Collagenase Type II, Worthington, CLS-2) with shaking until a single-cell suspension was obtained. During this incubation, the cells were dissociated at 10-min intervals by pipetting. To stop the collagenase activity, DMEM (GIBCO by Thermo Fisher Scientific, Waltham, MA USA, catalog n 61965059) containing 10% FBS (GIBCO by Thermo Fisher Scientific, Waltham, MA USA, catalog n 10270106) was added to the cell suspension, gently pelleted, and rinsed with PBS. The cells in PBS were then filtered through a 40 mm cell strainer (Corning Life Sciences, Corning, NY USA, catalog n 352340). Cells were washed and re-suspended in PBS. Then, embryonic ECs were isolated through MACS Technology by using nano-sized MicroBeads, following the manufacturer instructions. Particularly, a negative selection was performed using CD45 MicroBeads (Miltenyi Biotec, Bergisch Gladbach, DE, catalog n 130-052-301). CD45-negative cell fraction was then pelleted and incubated with CD31 MicroBeads (Miltenyi Biotec, Bergisch Gladbach, DE, catalog n 130-097-418) to isolate ECs (CD45−/CD31+ cell fraction).

### RNA extraction and quantitative real-time PCR analysis

RNA extraction and quantitative real-time PCR (qRT-PCR) analyses were performed as previously described [12]. Briefly, total RNA was extracted using PureLink RNA Mini Kit (Thermo Fisher Scientific, Waltham, MA USA) and 0.5–1 ug of total RNA was transcribed into complementary DNA (cDNA) by High-Capacity cDNA Reverse Transcription Kit (Thermo Fisher Scientific, Waltham, MA USA). qRT-PCR was performed using gene-specific TaqMan™ Gene Expression Assays (Thermo Fisher Scientific Waltham, MA USA). To detect FLVCR1a expression, specific primers and probes were designed using Primer Express Software Version 3.0 (Thermo Fisher Scientific, Waltham, MA USA). qRT-PCR was performed on a 7900HT Fast or QuantStudio™ 6 Flex Real-Time PCR System (Thermo Fisher Scientific, Waltham, MA USA), and the analyses were done using RQ Manager or QuantStudio Real-Time PCR software. Transcript abundance, normalized to 18 s messenger ribonucleic acid (mRNA) expression, is expressed as a fold increase over a calibrator sample.

### Histology, immunostainings, and morphometric analyses

Immunostainings of whole-mount embryos and retina samples were performed as described previously [12, 49]. Healthy organs (i.e., lung, liver) and LLC tumors were fixed in 4% PFA o/n at 4 °C, dehydrated, and embedded in paraffin or OCT. 5-μm-thick sections were stained with hematoxylin and eosin (H&E) or immunostained using specific antibodies. To visualize tumor blood vessels, 20-μm-thick cryosections were stained for CD31. The following primary antibodies were used: purified Rat Anti-Mouse CD31 (550274 BD Pharmingen™); Anti-Histone H3 (phospho S10) antibody (ab47297 Abcam); rabbit anti-NG2 chondroitin sulfate proteoglycan polyclonal antibody (AB5320 Merck Millipore). Network morphology was analyzed using specific plugins of Fiji, a distribution of the open-source software ImageJ [16, 53]. *Vascular area* was analyzed by measuring the ROIs given by CD31 + vessel area (lumen area included) and expressed as CD31/total area ratio. *Average Vessel Size* and *Vessels Distribution* (in terms of size) were obtained analyzing the same CD31 + ROIs with ImageJ. *Tumor vascularization* was calculated as number of vessels per area (mm^3^). Distance and variability of vessels was calculated by *ImageJ Nearest Neighbor Distances* (*NND) tool*. From this, the mean (*Average NND*) and the standard deviation (*Distribution*) were calculated to get information about the average distance to adjacent vascular segments and its variability. *Radial expansion* of the retinal vasculature was measured as the distance from the optic nerve to the vascular front. To calculate *Vascular Total Length*, number of *Branches*, *Average Vessel Perimeter*, and *Tortuosity*, images were skeletonized and analyzed with the ImageJ plugin *Skeleton length tool*. The complete FIJI scripts are available on request. Confocal image acquisition was performed with a Leica TCS SP8 confocal system (Leica Microsystems) with a PL APO 20 ×/0.75 CS2 or HC PL APO 40 ×/1.30 OIL CS2 objectives. Microscopic analysis of histological sections was done with Automated Upright Microscope Leica DM6 B. Zebrafish and mouse embryos images were acquired using Leica MZ10 F modular stereo microscope with 8 ×–80 × magnification and 375 Lp/mm high resolution with fluorescent illumination.

### Statistics

Statistical comparisons were conducted in Prism (GraphPad Software, Inc., La Jolla, CA). Results are expressed as mean ± SEM (standard error mean). Whenever two groups were compared, (1) Normally distributed data were tested for differences with a two-tailed unpaired one-sample t test; (2) Non-normally distributed data were tested with a Mann–Whitney *U* test. The level of significance was set at *p < 0.05, **p < 0.01, ***p < 0.001, ****p < 0.0001.

## Supplementary Information

Below is the link to the electronic supplementary material.

Supplementary file1 (DOCX 2217 KB)
