# Supplementary material for: Endothelial cells require functional FLVCR1a during developmental and adult angiogenesis
Source: Angiogenesis. 2023 Jan 11;26(3):365–84. doi: 10.1007/s10456-023-09865-w (PMC10328904; doi:10.1007/s10456-023-09865-w)
Supplement: Supplementary file 1 — Supplementary file1 (DOCX 2217 KB) [file 10456_2023_9865_MOESM1_ESM.docx]

**
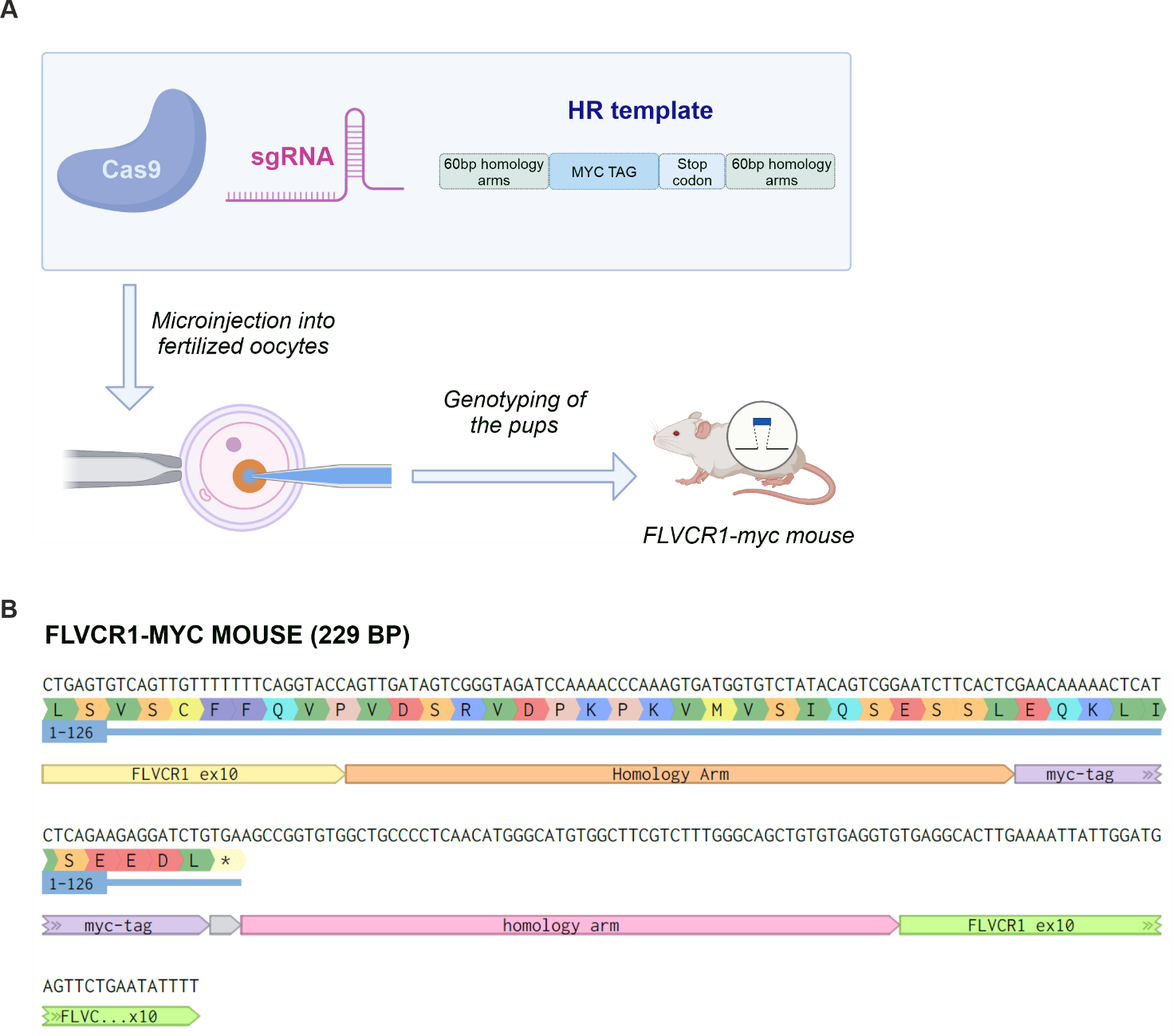
**

**Figure S1 Generation of FLVCR1-myc mouse. (A)** Schematic representation of FLVCR1-myc mouse generation through CRISPR-Cas9 technique. The myc-tag was introduced by homologous recombination (HR) at the terminal end of the *Flvcr1a* coding region, using a specific sgRNA and a replacement single stranded DNA. **(B)** Sequencing results from FLVCR1-myc mouse genomic DNA. The image shows that the myc-tag is in frame with the *Flvcr1* gene.

**
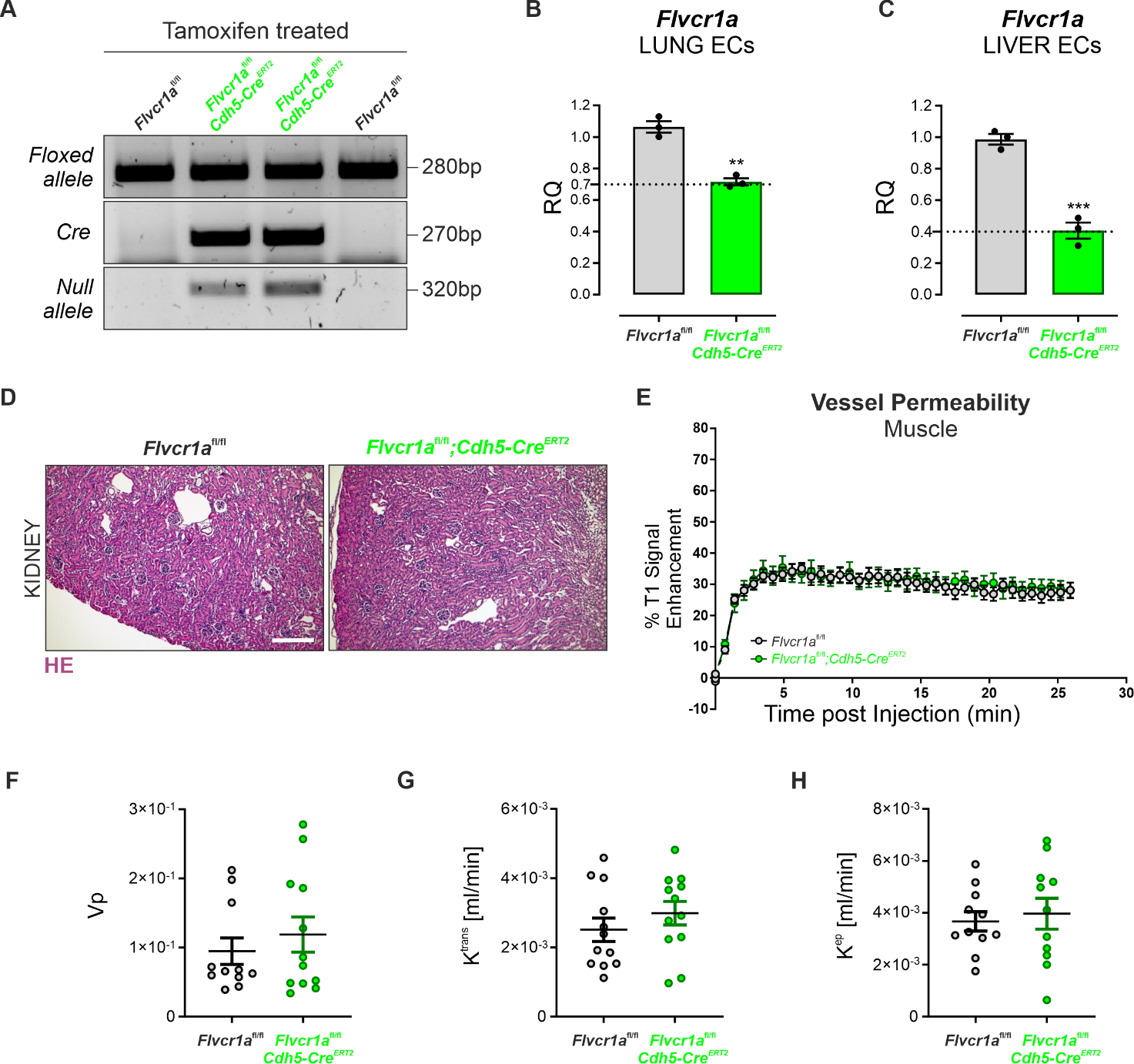
**

**Figure S2 Endothelial *Flvcr1a* targeting in quiescent vasculature does not affect tissue morphology and vascular permeability.** (**A**) Representative PCR products from DNA analysis on *Flvcr1a*^fl/fl^;*Cdh5-Cre*^ERT2^ mice and control *Flvcr1a*^fl/fl^ mice after tamoxifen injection. Deletion of the first exon of *Flvcr1a* gene mediated by Cre recombinase gave rise to a band referred to as the ‘‘null allele”. Specific primers allowed the distinction of the floxed (280 bp) and null (320 bp) allele of *Flvcr1a*. (**B**-**C**) qRT-PCR analysis showing *Flvcr1a* mRNA levels in ECs isolated from *Flvcr1a*^fl/fl^ and *Flvcr1a*^fl/fl^;*Cdh5-Cre*^ERT2^ (B) lung and (C) liver. (**D**) Representative histological sections of kidney tissue from *Flvcr1a*^fl/fl^ and *Flvcr1a*^fl/fl^;*Cdh5-Cre*^ERT2^ mice. Tissue sections stained with hematoxylin and eosin to look at the cellular morphology. Scale bar: 200 µm. (**E-H**) Gadolinium-based DCE-MRI analysis to evaluate vascular permeability. The (E) contrast agent enhancement curve, the (F) total vascular volume and the vascular (G-H) perfusion parameters (i.e. K^trans^, K^ep^) in *Flvcr1a*^fl/fl^ and *Flvcr1a*^fl/fl^;*Cdh5-Cre*^ERT2^ muscles are shown. HE, hematoxylin/eosin DCE-MRI, Dynamic Contrast-Enhanced Magnetic Resonance Imaging, Vp, vascular volume. Data are representative of at least 3 independent experiments and are expressed as mean ± SEM. **p<0.01; ***p<0.001; ns, not significant. For statistical analyses an unpaired Student’s t-test was used.

**
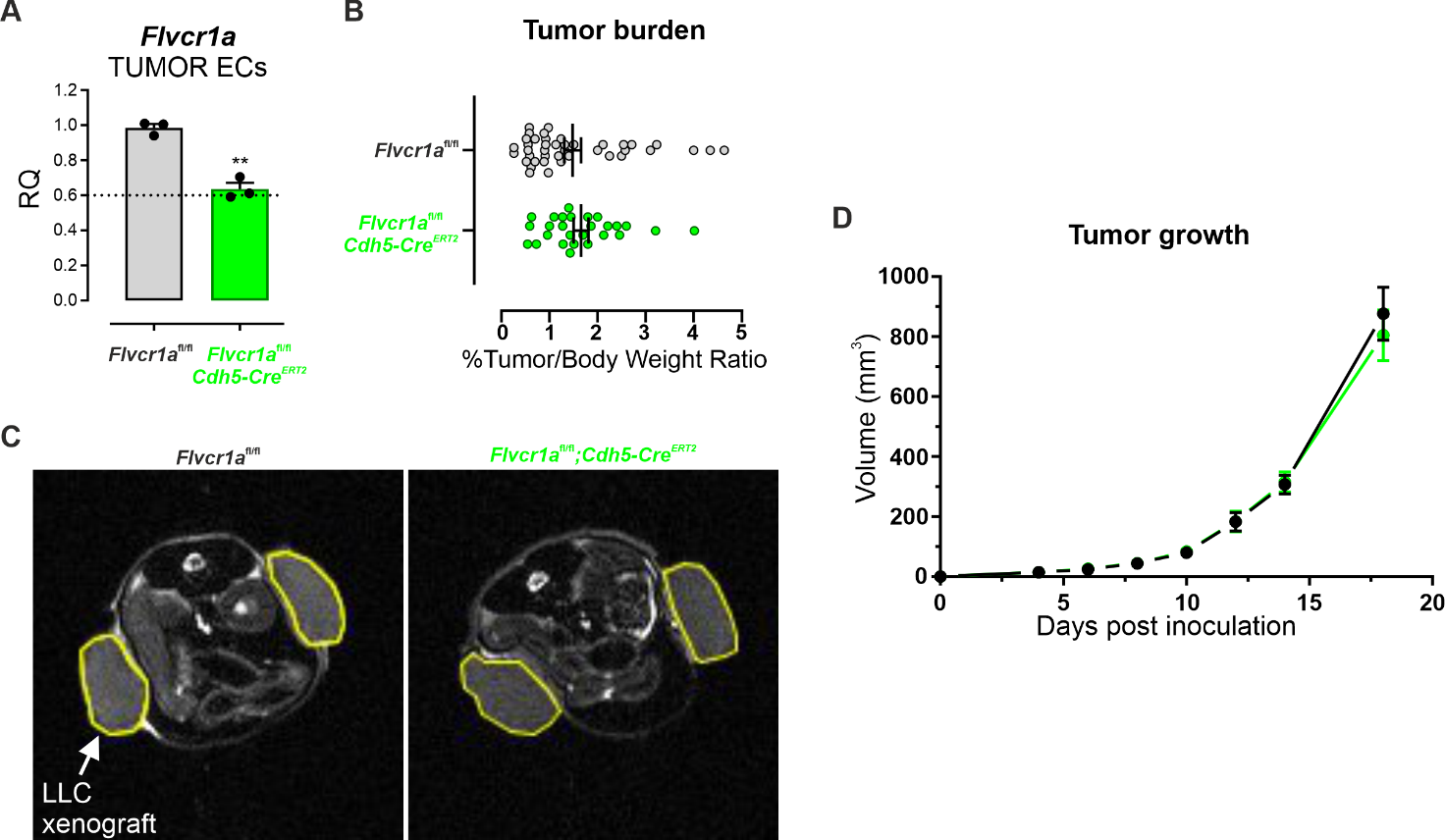
**

**Figure S3 Endothelial *Flvcr1a* targeting does not modulate tumor growth.** (**A**) qRT-PCR analysis showing *Flvcr1a* mRNA levels in ECs isolated from *Flvcr1a*^fl/fl^ and *Flvcr1a*^fl/fl^;*Cdh5-Cre*^ERT2^ LLC-xenografts. (**B**) Tumor burden in *Flvcr1a*^fl/fl^ and *Flvcr1a*^fl/fl^;*Cdh5-Cre*^ERT2^ mice, expressed as % of tumor/body weight ratio. n=27 to 41 tumors. (**C**) Representative T2w MRI images of *Flvcr1a*^fl/fl^ and *Flvcr1a*^fl/fl^;*Cdh5-Cre*^ERT2^ LLC-xenografts mice. (**D**) MRI-based analysis of tumor volume at multiple time points. n=6 to 10 tumors. Data are are expressed as mean ± SEM. **p<0.01. For statistical analyses an unpaired Student’s t-test was used.
